# Inactivation of a Novel FGF23 Regulator, FAM20C, Leads to Hypophosphatemic Rickets in Mice

**DOI:** 10.1371/journal.pgen.1002708

**Published:** 2012-05-17

**Authors:** Xiaofang Wang, Suzhen Wang, Changcheng Li, Tian Gao, Ying Liu, Afsaneh Rangiani, Yao Sun, Jianjun Hao, Anne George, Yongbo Lu, Jay Groppe, Baozhi Yuan, Jian Q. Feng, Chunlin Qin

**Affiliations:** 1 Department of Biomedical Sciences, Texas A&M Health Science Center, Baylor College of Dentistry, Dallas, Texas, United States of America; 2 Department of Prosthodontics, School of Stomatology, Harbin Medical University, Harbin, Heilongjiang, China; 3 Department of Craniofacial Sciences, Division of Orthodontics, School of Dental Medicine, University of Connecticut Health Center, Farmington, Connecticut, United States of America; 4 Department of Oral Biology, University of Illinois at Chicago, Chicago, Illinois, United States of America; 5 Department of Medicine, University of Wisconsin, Madison, Wisconsin, United States of America; 6 Geriatric Research Education and Clinical Centers, Madison, Wisconsin, United States of America; Indiana University School of Medicine, United States of America

## Abstract

Family with sequence similarity 20,-member C (FAM20C) is highly expressed in the mineralized tissues of mammals. Genetic studies showed that the loss-of-function mutations in *FAM20C* were associated with human lethal osteosclerotic bone dysplasia (Raine Syndrome), implying an inhibitory role of this molecule in bone formation. However, *in vitro* gain- and loss-of-function studies suggested that FAM20C promotes the differentiation and mineralization of mouse mesenchymal cells and odontoblasts. Recently, we generated *Fam20c* conditional knockout (cKO) mice in which *Fam20c* was globally inactivated (by crossbreeding with Sox2-Cre mice) or inactivated specifically in the mineralized tissues (by crossbreeding with 3.6 kb Col 1a1-Cre mice). *Fam20c* transgenic mice were also generated and crossbred with *Fam20c* cKO mice to introduce the transgene in the knockout background. *In vitro* gain- and loss-of-function were examined by adding recombinant FAM20C to MC3T3-E1 cells and by lentiviral shRNA–mediated knockdown of FAM20C in human and mouse osteogenic cell lines. Surprisingly, both the global and mineralized tissue-specific cKO mice developed hypophosphatemic rickets (but not osteosclerosis), along with a significant downregulation of osteoblast differentiation markers and a dramatic elevation of fibroblast growth factor 23 (FGF23) in the serum and bone. The mice expressing the *Fam20c* transgene in the wild-type background showed no abnormalities, while the expression of the *Fam20c* transgene fully rescued the skeletal defects in the cKO mice. Recombinant FAM20C promoted the differentiation and mineralization of MC3T3-E1 cells. Knockdown of FAM20C led to a remarkable downregulation of DMP1, along with a significant upregulation of FGF23 in both human and mouse osteogenic cell lines. These results indicate that FAM20C is a bone formation “promoter” but not an “inhibitor” in mouse osteogenesis. We conclude that FAM20C may regulate osteogenesis through its direct role in facilitating osteoblast differentiation and its systemic regulation of phosphate homeostasis via the mediation of FGF23.

## Author Summary

A recent study demonstrated that the inactivating mutations in the *FAM20C* gene were associated with lethal osteosclerotic bone dysplasia characterized by a generalized hardening of all bones; this observation implied an inhibitory role of FAM20C during bone formation. However, *in vitro* studies revealed a contradictory finding that FAM20C accelerated the differentiation of cells forming the mineralized tissues. Here we generated *Fam20c* conditional knockout (cKO) mice, in which the gene was inactivated either in all tissues or specifically in the mineralized tissues. We also generated recombinant FAM20C protein and *Fam20c* transgenic mice. The cKO mice did not mimic the human skeleton abnormalities of osteosclerotic bone dysplasia, but exhibited rickets (softer bone) along with a significant reduction of serum phosphate level and a remarkable elevation of serum FGF23, a hormone known to promote phosphate wasting. A number of differentiation markers of the bone-forming cells were downregulated in the cKO mice. Recombinant FAM20C promoted the differentiation of mouse preosteoblasts. Introducing the *Fam20c* transgene did not lead to any abnormalities but rescued the bone defects of the cKO mice. Taken together, we conclude that FAM20C promotes the differentiation of osteoblast lineages and regulates phosphate homeostasis via the mediation of FGF23.

## Introduction

FAM20C is a member of the “family with sequence similarity 20”. In mammals, this evolutionarily conserved protein family consists of three members: FAM20A, FAM20B and FAM20C. FAM20A was originally observed in the lung and liver and displays obvious differential expression in hematopoietic cells undergoing myeloid differentiation [1]. A viral mRNA transgenic mouse line with an accidental deletion of a 58-kb fragment in chromosome 11E1 encompassing part of the *Fam20a* gene and its upstream region showed growth disorder [2]. Recently, it was found that FAM20A is also expressed in ameloblasts and its mutations are associated with human amelogenesis imperfecta and gingival hyperplasia syndrome [3]. More recently, FAM20B was shown to be involved in cartilage matrix production and the ultimate regulation on the timing of skeletal development [4]. FAM20C is highly expressed in the mineralized tissues and identified as the causal gene for lethal osteosclerotic bone dysplasia (Raine Syndrome, OMIM 259775) [1], [5]–[7]. Given the high level of conservation in the C-terminal domains among the three FAM20 members and the their roles observed in the hard tissues, it is tempting to speculate that this evolutionarily conserved family might be a new cluster of molecules performing important functions in the development of the mineralized tissues.

Mouse FAM20C, also known as “dentin matrix protein 4” (DMP4) [5], contains 579 amino acid residues, including a putative 26-amino acid signal peptide at the N-terminus. A C-terminal region of approximately 350 amino acids (corresponding to residue^218^-residue^569^ in the mouse FAM20C sequence) has been named the “conserved C-terminal domain” (CCD), which is highly conserved among different species [1].

In a previous study, we systematically analyzed the expression and distribution of FAM20C in mouse bone and tooth using *in situ* hybridization (ISH) and immunohistochemistry (IHC) methods [7], which showed that FAM20C was highly expressed in the mineralized tissues; it was detected in the osteoblasts/osteocytes, odontoblasts, ameloblasts, and cementoblasts, as well as in the matrices of bone, dentin, and enamel. FAM20C was also detected in the epithelium of early-stage tooth germs and in the chondrogenic cells of long bones. The high expression levels of FAM20C in the mineralized tissues strongly suggest that it may play an important role in the formation and/or mineralization of these tissues.

Hao *et al.* showed that overexpression of mouse FAM20C accelerated the odontoblast differentiation process and silencing this molecule by siRNA inhibited cell differentiation, implying that this protein may be a factor promoting odontoblast differentiation [5]. Subsequently, Simpson *et al.* reported that the loss-of-function mutations in the *FAM20C* gene were associated with lethal/non-lethal osteosclerotic bone dysplasia (Raine Syndrome) [6], [8], an autosomal recessive disorder characterized by a generalized increase in the density of all bones; these data indicated that FAM20C might be a down-regulator of biomineralization, which apparently contradicts the mineralization-promoting properties of FAM20C observed by Hao *et al.*

In this study, we sought to determine the biological functions of FAM20C via generation and characterization of *Fam20c* conditional knockout (cKO) mice. Our data showed remarkable skeletal defects, along with a significant reduction of serum phosphate and a dramatic elevation of serum fibroblast growth factor 23 (FGF23) in the homozygous *Fam20c* cKO mice. The phenotypic profiles of the *Fam20c*-deficient mice resemble those of hereditary hypophosphatemic rickets in humans and rodents resulting from mutations in molecules affecting the regulation of FGF23 [9]–[15].

## Results

### Validation of FAM20C inactivation in conditional knockout mice

**Figure 1:**
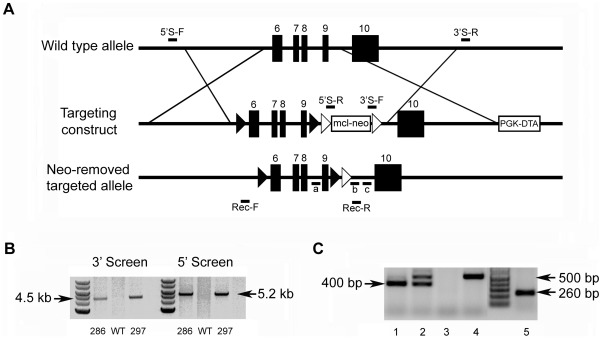
Generation of the *Fam20c* floxed alleles and *Fam20c* null alleles. (A) Targeting construct. Dark boxes: Exons; dark triangles: loxP sites. A mcl-neo cassette flanked by Frt sites (white triangles) was inserted between exons 9 and 10. A PGK-DTA cassette was downstream to the 3′ homologous arm. The mcl-neo cassette was removed from the targeted allele after correct targeting. (B) PCR screening for targeted ES clones. The correct targeting was confirmed by PCR using 5′ screen primers (5′S-F and 5′S-R) and 3′ screen primers (3′S-F and 3′S-R). The correct targeting produced a 5.2 kb fragment for the 5′ screening, and a 4.5 kb fragment for the 3′ screen. WT and random insertion had no PCR products. Two correctly targeted ES clones (Clone 286 and Clone 297) were identified and both went through germline transmission. (C) Genotyping strategy. The alleles were genotyped by PCR using a mixture of three primers: “a”, “b” and “c” (see Figure 1A). The primers “a” and “b” produced a 400 bp fragment for the floxed allele. The primers “a” and “c” produced a 500 bp fragment for WT allele. The null alleles did not produce any PCR products due to the loss of the binding sequences for primer “a”. The Cre-loxP recombination was confirmed by PCR using primers Rec-F and Rec-R. A 260 bp fragment was produced for the null allele, but no PCR products for the WT allele. Lane 1 indicated the *Fam20c* ^flox/flox^ genotype. Lane 2 referred to the *Fam20c* ^flox/+^. Lane 3 indicated the *Fam20c* ^Δ/Δ^. Lane 4 demonstrated the WT. Lane 5 showed the Cre-LoxP recombination on *Fam20c* floxed allele.

The mouse *Fam20c* gene consists of 10 exons and spans approximately 55-kb. To generate a conditional knockout allele for *Fam20c*, we constructed a targeting vector with loxP sites floxing exons 6∼9 which are highly conserved across species (Figure 1A); a number of mutations were identified in this region of the human *FAM20C* gene in patients with lethal osteosclerotic bone dysplasia [6]. The correct targeting events were confirmed by polymerase chain reaction (PCR) screening, and the presence of 5′ and 3′ loxP sites was determined by PCR product sequencing. Two correctly targeted ES cell clones were identified (Figure 1B, Clones 286 and 297), and both went through germline transmission. F1 *Fam20c* ^flox/+^ heterozygous mice were crossbred with Sox2 promoter-Cre transgenic mice to generate “*Sox2-Cre-Fam20c* ^Δ/Δ^” mice, in which exons 6∼9 were removed from both alleles of the *Fam20c* gene in the epiblasts at post coitum day 6.5 (E6.5). The presence of the floxed alleles and the absence of exons 6∼9 in the null alleles were confirmed by PCR genotyping (Figure 1C).

**Figure 2:**
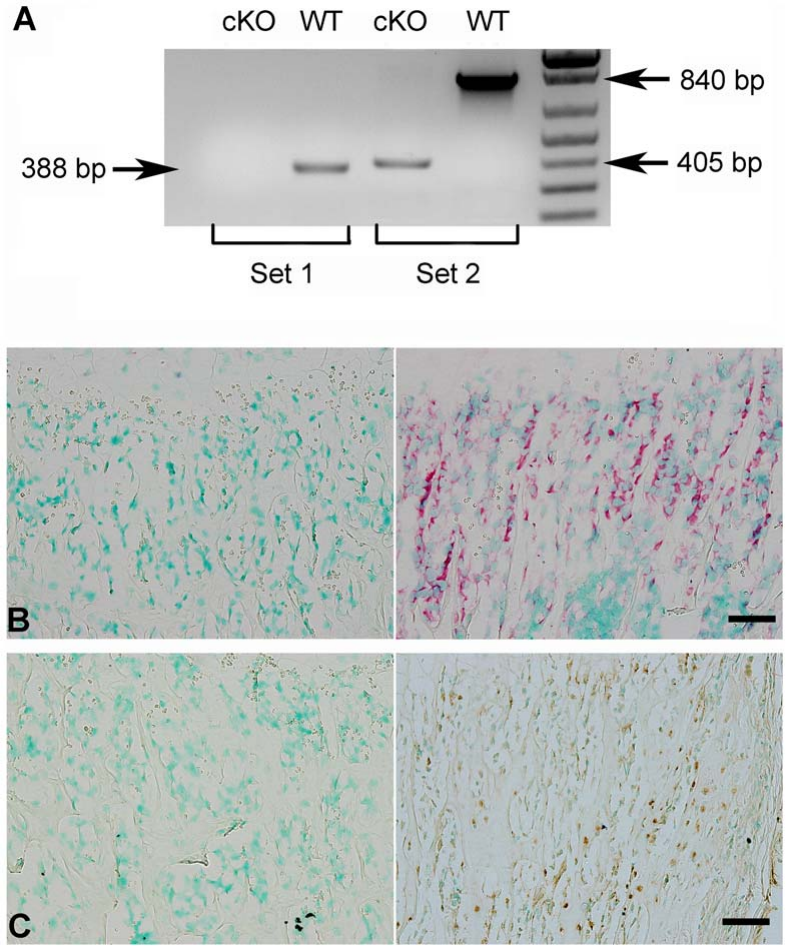
Validation of FAM20C inactivation. (A) RT-PCR was performed with the cDNAs reversely transcripted from the total RNAs extracted from the femurs of 3-week-old *Sox2-Cre-Fam20c* ^Δ/Δ^ (cKO) mice and WT littermates, using two sets of primers: Set 1 primers (the forward primer was in exon 4, and the reverse in exon 7) produced a 388 bp fragment for WT mice, and gave rise to no product from the cKO mice (due to exon 7 ablation); Set 2 primers (the forward in exon 4, and the reverse in exon 10) produced a 840 bp fragment for the WT mice, and a 405 bp fragment for the cKO mice. (B) ISH on femurs. The osteoblasts in the trabecular bone area of 3-week-old cKO mice had negative staining for *Fam20c* mRNA (left), in contrast with the strong staining in the WT mice (right). (C) IHC on femurs. The osteoblasts and osteocytes of cKO mice (left) showed negative staining for FAM20C protein, while positive staining was observed in the WT littermates (right). Scale bars: 50 µm.

The lack of *Fam20c* mRNA in the *Sox2-Cre-Fam20c* ^Δ/Δ^ mice was shown by reverse transcription PCR (RT-PCR) performed with two sets of primers using mRNA extracted from the long bones (Figure 2A), as well as by in situ hybridization (ISH) carried out on the long bones (Figure 2B). The lack of FAM20C protein was determined by immunohistochemistry (IHC) analyses (Figure 2C) performed on the long bones using an affinity-purified anti-FAM20C polyclonal antibody [7].

Both male and female *Sox2-Cre-Fam20c* ^Δ/Δ^ (homozygous cKO) mice are infertile, while the *Sox2-Cre-Fam20c* ^Δ/+^ (heterozygous cKO) mice have normal fertility. The *Fam20c* ^flox/flox^ mice and the heterozygous cKO mice did not demonstrate any phenotypic changes compared with their wild type (WT) littermates (data not shown), while the homozygous cKO mice displayed remarkable skeletal defects, indicating that the haploinsufficiency of *Fam20c* has no significant effects on the bone formation. We also bred the *Fam20c* ^flox/flox^ mice with the 3.6 kb *Col 1a1-Cre* mice to generate *Col1a1-Cre-Fam20c* ^Δ/Δ^ mice, which displayed skeletal defects similar to those observed in the *Sox2-Cre-Fam20c* ^Δ/Δ^ mice. In this report, we described in detail the analyses of phenotypic changes in the *Sox2-Cre-Fam20c* ^Δ/Δ^ mice while the X-ray and histology data of the long bone from the *Col1a1-Cre-Fam20c* ^Δ/Δ^ mice were included in one set of the figures to show the similarity between the global and mineralized tissue-specific cKO mice. The data regarding the *Fam20c* cKO mice refer to the analyses of the *Sox2-Cre-Fam20c* ^Δ/Δ^ mice unless otherwise stated.

### Inactivation of FAM20C leads to skeletal defects

#### Inactivation of FAM20C leads to growth retardation

**Figure 3:**
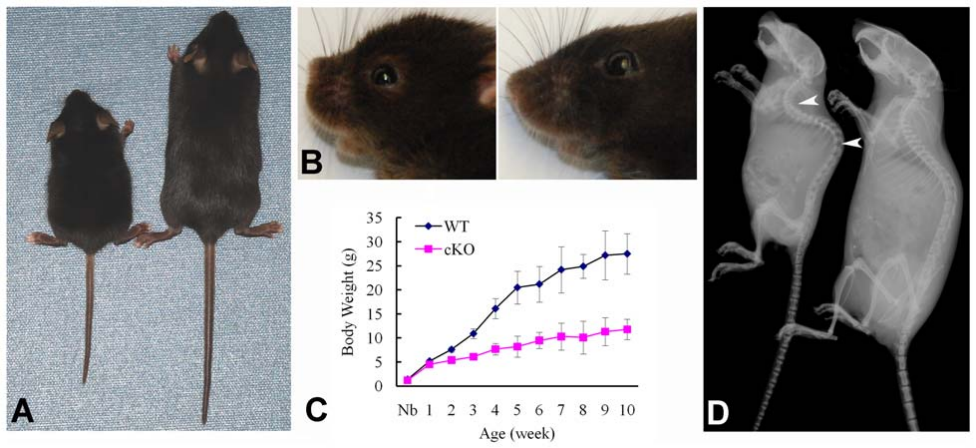
Gross defects in the *Sox2-Cre-Fam20c*-cKO mice. (A) The 4-week-old *Sox2-Cre-Fam20c* ^Δ/Δ^ (cKO) mouse on the left was smaller compared with the WT littermate on the right. (B) The 4-week-old cKO mouse (left) had flat face (undeveloped nose, a typical manifestation of rickets) compared with the WT littermate (right). (C) Body weight monitoring from newborn (Nb) to postnatal 10 weeks revealed significant growth retardation in the cKO mice. (D) Plain X-ray examination of a 5-month-old cKO mouse (left) revealed smaller skeleton, hypomineralization and distorted spine (arrowheads) compared with its WT littermate (right).

**Figure 4:**
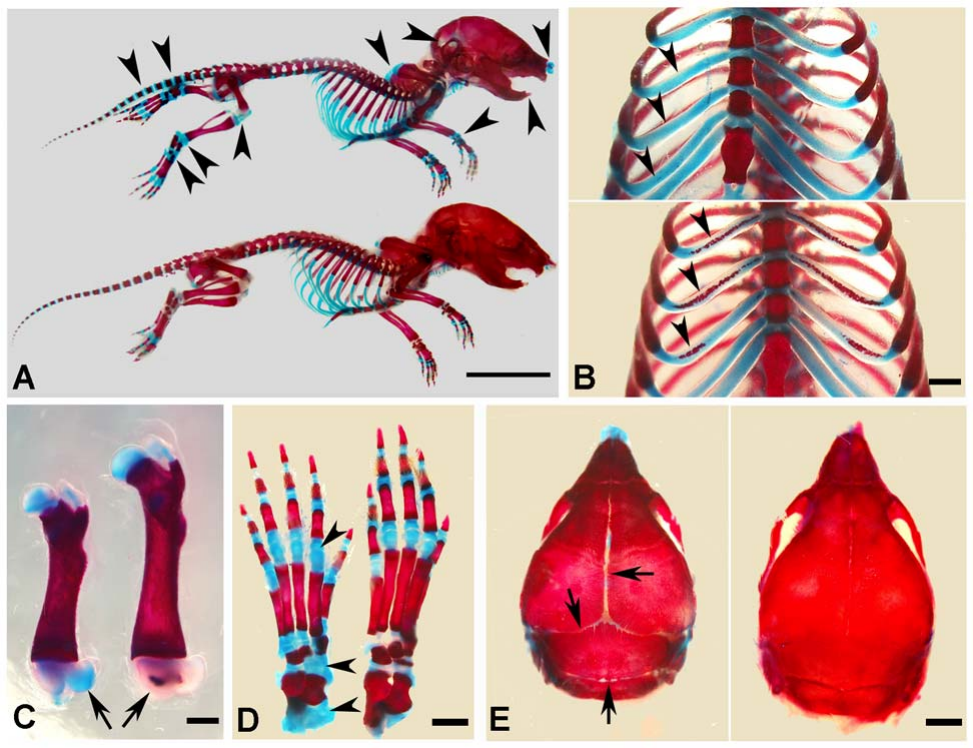
Alizarin red/alcian blue staining of the skeleton in the 1-week-old *Sox2-Cre-Fam20c*-cKO mice. (A) The *Sox2-Cre-Fam20c* ^Δ/Δ^ (cKO) mice (upper) showed smaller size and remarkably delayed ossification (more blue staining) in all bones (arrowheads) when compared with WT littermates (lower). (B) The rib cartilages of cKO mice (upper) stained blue, while those of their WT littermates (lower) showed broad areas with the red staining indicating ossification (arrowheads), suggesting an aberrant endochondral ossification in the cKO mice. (C) The femur of cKO mice (left) had shorter length and delayed secondary ossification center (arrow, blue stained), in contrast with the red secondary ossification center of their WT littermates (right). (D) The ossification centers in the carpus of the cKO mice (left) had more blue-stained areas than that of the WT littermates (right). (E) The skull of the cKO mice (left) had smaller size and delayed suture closure (arrows), when compared with the WT littermates (right), indicating that the intramembranous ossification was disturbed in the cKO mice. Scale bars: 1 cm in A, 2 mm in E, 1 mm in B–D.

At the gross level, the 4-week-old *Sox2-Cre-Fam20c* ^Δ/Δ^ *mice* demonstrated prominent dwarfism and flat faces (Figure 3A and 3B). The body weight of the *Fam20C*-deficient mice was significantly lower than that of their WT littermates, indicating retardation in the growth of the mutant mice (Figure 3C). X-ray examination showed that the 5-month-old *Sox2-Cre-Fam20c* ^Δ/Δ^ mice had smaller skeletons, lower levels of mineralization and distorted spines (Figure 3D). The Alizarin Red/Alcian Blue staining of the skeletons from 1-week-old mice revealed that the *Sox2-Cre-Fam20c* ^Δ/Δ^ mice had smaller stature (Figure 4A), delayed ossification in the ribs (Figure 4B), long bones (Figure 4C) and carpus (Figure 4D), along with smaller skulls and delayed cranial suture closure (Figure 4E). These observations indicated that the *Fam20c*-deficient mice had extensive defects in both endochondral and intramembranous ossifications, resulting in a generalized hypomineralization in the axial skeleton and craniofacial complex.

#### Inactivation of FAM20C leads to rickets/osteomalacia

**Figure 5:**
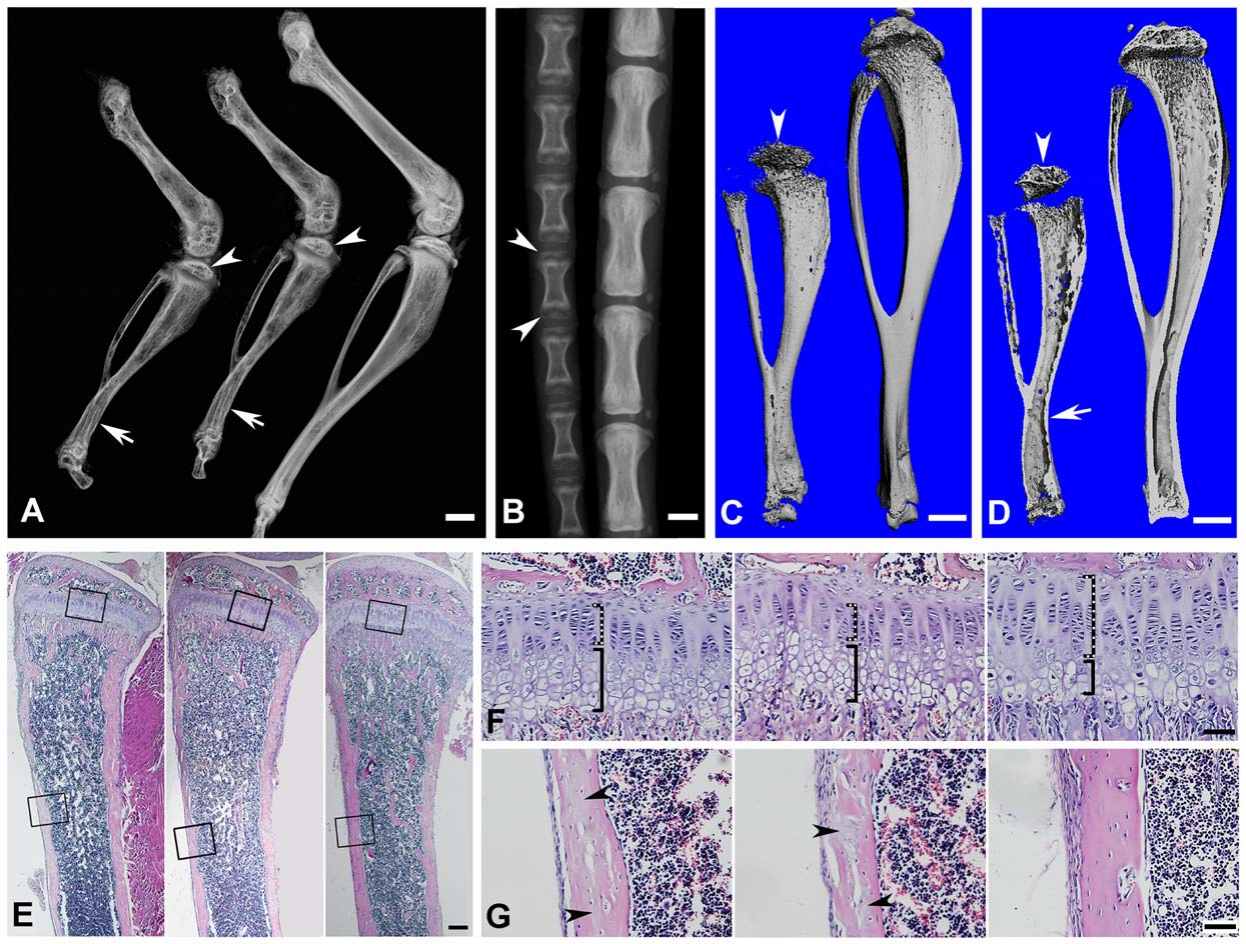
Bone defects in the 6-week-old *Fam20c*-cKO mice revealed by X-ray and histology. (A) Plain X-ray of the hinder legs. The tibia of the *Sox2-Cre-Fam20c* ^Δ/Δ^ (global cKO) mice (left) showed shorter length, hypomineralization, thinner cortical bone (arrow) and underdeveloped secondary ossification center (arrowhead) compared with their WT littermates (right). The long bones of the *Col1a1-Cre-Fam20c* ^Δ/Δ^ (mineralized tissue-specific cKO) mice (middle image) showed defects very similar to those of the global-cKO mice. (B) Plain X-ray of the tail of *Sox2-Cre-Fam20c* ^Δ/Δ^ mice (left) showed hypomineralization, shorter length, and underdeveloped secondary ossification centers (arrowheads), compared with the WT littermates (right). (C,D) Micro-CT analyses. The tibia of the *Sox2-Cre-Fam20c* ^Δ/Δ^ mice (left) showed shorter length, thinner cortical bone (arrow), more porous areas on both the outer and inner surfaces (indicating more hypominerlized areas) and smaller secondary ossification centers (arrowheads), compared with their WT littermates (right). (E) H&E staining of the sagittal sections of tibias. Tibias of the *Sox2-Cre-Fam20c* ^Δ/Δ^ mice (left image) and *Col1a1-Cre-Fam20c* ^Δ/Δ^ mice (middle image) were smaller and underdeveloped compared with the WT littermates (right image). (F) Higher magnification of the metaphysis areas in E showed that the growth plates of the global cKO mice (left image) and mineralized tissue-specific cKO mice (middle image) had a thinner zone of proliferative chondrocytes (dashed lines) and a wider zone of hypertrophic chondrocytes (solid lines) than the WT littermates (right image). (G) Higher magnification of the cortical bone areas in E showed that the cortical bone of the global cKO mice (left image) and mineralized tissue-specific cKO mice (middle image) had more osteoids (grey areas indicated by arrowheads) compared with the WT littermates (right image). Scale bars: 1 mm in A–D, 200 µm in E, 50 µm in F and G.

Plain X-ray examination did not reveal obvious skeletal abnormalities in the *Sox2-Cre-Fam20c* ^Δ/Δ^ mice at birth (data not shown). However, the bone defects in the *Sox2-Cre-Fam20c* ^Δ/Δ^ mice could be easily identified by X-ray radiography after postnatal 4 days, and the phenotypic changes in the skeleton became more profound as the animals aged. Multiple fractures were often seen in the *Sox2-Cre-Fam20c* ^Δ/Δ^ mice after the age of 3 weeks (data not shown). At the age of 6 weeks, X-ray radiography revealed lower density in the bones of the *Sox2-Cre-Fam20c* ^Δ/Δ^ (global cKO) mice (indicating a lower level of mineralization), along with a delay of the secondary ossification centers in the epiphysis in the long bones (left image in Figure 5A) and vertebrae (left image in Figure 5B). Similar bone defects were observed in the 3.6 kb Col1a1-Cre induced mineralized tissue-specific cKO mice (middle image in Figure 5A). In addition to the delayed ossification, micro-CT (μ-CT) analyses showed increased porosity in the diaphyses and malformed epiphyses of the global cKO mice (Figure 5C and 5D). The appearance of more porous areas in both the outer and inner bone surfaces of the mutant mice seen in the μ-CT radiogram was a result of increased areas of hypomineralization. The μ-CT quantitative analyses showed a significantly lower mineral Apparent Density and Material Density in the tibia midshaft region of the 3-week- and 6-week-old global cKO mice (Table 1).

**Table 1:** Quantitative μ-CT analyses of the cortical bone (the midshaft region) of the tibias from 3-week-old and 6-week-old WT and *Fam20c*-cKO mice.

Variables	3-week-old		6-week-old	
(Mean ± SD)	WT (n = 6)	cKO (n = 6)	WT (n = 6)	cKO (n = 6)
BV/TV	0.91±0.12	0.70±0.05*	0.96±0.11	0.91±0.12
Apparent density (mg/cm^3^)	715±12	505±16*	924±15	708±13*
Material density (mg/cm^3^)	831±13	681±15*	1021±18	827±16*

BV/TV: ratio of bone volume (BV) to total volume (TV); Apparent density: average mineral density within the segmented area of all voxels thresholded as bone; Material density: average mineral density of all voxels segmented as bone including voids;

*: : significant difference from WT mice (p<0.05).

**Figure 6:**
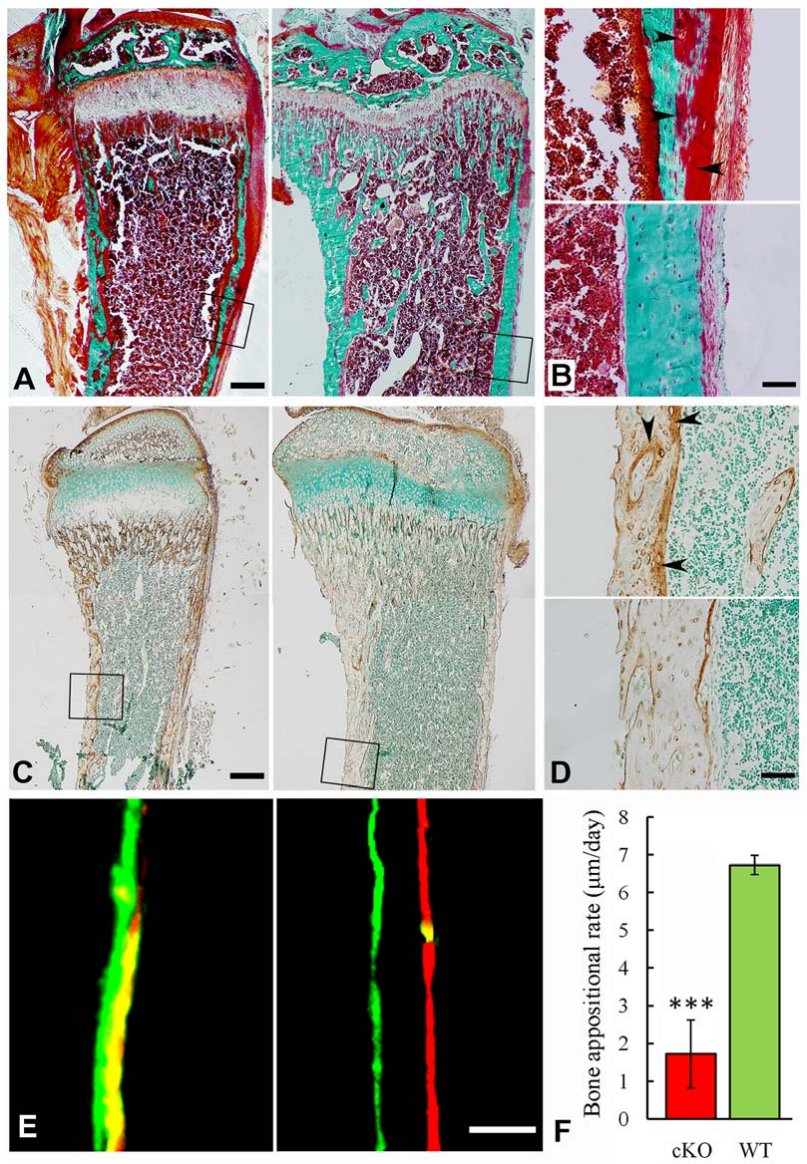
Reduced mineralization level in the *Sox2-Cre-Fam20c*-cKO mice. (A) Goldner-Masson trichrome staining of sagittal sections from the undecalcified tibias of the cKO mice (left) and their WT littermates (right). The cKO mice showed more red staining (unmineralized osteoid) and less green staining (mineralized bone) compared with the WT. (B) Higher magnification of the cortical bone areas in A showed that the cortical bone of the cKO mice (upper) had more osteoid (red stained areas indicated by arrowheads), compared to the WT (lower). (C) IHC staining against biglycan on the sagittal sections of tibias from 3-week-old cKO mice (left) and their WT littermates (right). Note that the cKO mice had more biglycan. (D) Higher magnification of the cortical bone areas in C showed that the cortical bone of cKO mice (upper) had more biglycan (arrowheads), compared with the WT (lower). (E) Double fluorescence labeling of the tibia from 6-week-old cKO mice (left) and WT littermates (right). The first injection (calcein) produced a green label, while the second injection (Alizarin Red) gave rise to a red label. The distance between the green and red labeling indicated the mineral deposition in the period between the two injections (7 days). The tibia cortical bone of the cKO mice (left) showed narrower distance and blurry boundary between the two labels compared with the WT (right). (F) The quantitative measurements of the distance between the two injections revealed a significantly lower mineral deposition rate in the cKO mice compared with their WT littermates. ***P<0.005. Scale bars: 200 µm in A and C, 50 µm in B, D and E.

Histological analyses with H&E staining showed that the long bone of the global cKO mice had a thinner cortical bone along with a reduced proliferative zone and an enlarged hypertrophic zone in the growth plates (left image in Figure 5E–5G). Similar histological defects were observed in the Col1a1-Cre induced mineralized tissue-specific cKO mice (middle image in Figure 5E–5G). Goldner's Masson Trichrome staining showed that the diaphysis region of the tibia in the *Sox2-Cre-Fam20c* ^Δ/Δ^ mice had more osteoid/hypomineralized areas (stained red) in the cortical bones (Figure 6A and 6B). The broad areas with larger amounts of osteoid/hypomineralized tissues in the *Sox2-Cre-Fam20c* ^Δ/Δ^ showed a remarkable increase in the immunostaining for biglycan (Figure 6C and 6D) [16]. The double fluorochrome labeling analyses showed that the *Sox2-Cre-Fam20c* ^Δ/Δ^ mice had a significantly lower mineral deposition rate compared with their WT littermates (Figure 6E and 6F). Taken together, the phenotypic changes in the skeletons of the global and mineralized tissue-specific cKO mice were consistent with a diagnosis of rickets/osteomalacia.

#### Inactivation of FAM20C leads to defects in the growth plate

**Figure 7:**
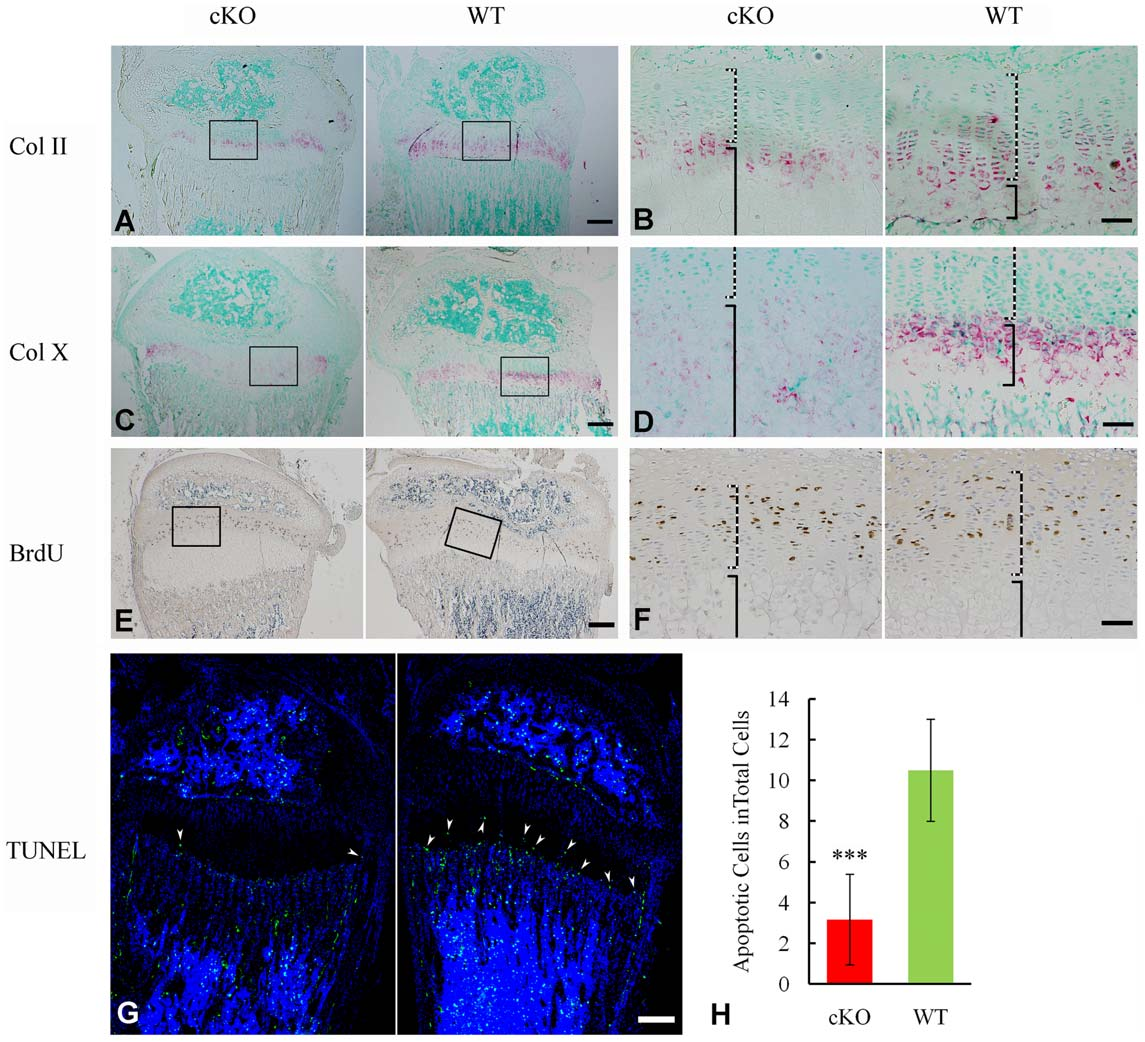
Growth plate defects in the 3-week-old *Sox2-Cre-Fam20c*-cKO mice. (A) ISH staining of Col II on the sagittal sections of tibia showed less Col II in the growth plate of cKO mice. The pink/red color indicates positive ISH staining. (B) Higher magnification of the boxed area in A revealed less Col II in both the proliferative chondrocytes (dashed lines) and hypertrophic chondrocytes (solid lines) in the growth plate of the cKO mice compared with WT littermates. (C) ISH staining of Col X showed lower expression in the growth plate of the cKO mice. (D) Higher magnification of the boxed area in C revealed less Col X in the hypertrophic chondrocytes (solid lines) of the cKO mice compared with WT. (E) BrdU staining on the sagittal sections of tibia. (F) Higher magnification of the boxed area in E revealed fewer proliferating cells in the zone of proliferative chondrocytes (dashed lines) of the cKO mice compared with WT. (G) TUNEL assay revealed fewer apoptotic cells in the zone of hypertrophic chondrocytes (arrowheads) of the cKO mice (left) compared with WT littermates (right). Counter stained with DAPI. (H) Statistical counting of apoptotic cells in G showed significantly less number in the growth plate of the cKO mice. *** P value<0.001. Scale bars: 200 µm in A, C, E and G. 50 µm in B, D and F.

X-ray analyses revealed hypomineralized metaphysis and growth plate in the *Sox2-Cre-Fam20c* ^Δ/Δ^ mice (Figure 5A–5D). Histological examination displayed an altered thickness of the proliferative and hypertrophic zones in the long bones of the *Sox2-Cre-Fam20c* ^Δ/Δ^ mice (Figure 5F). To determine the molecular changes associated with the growth plate defects, we examined the differentiation, proliferation and apoptosis of the chondrocytes in the growth plates. *In situ* hybridization (ISH) analyses showed that type IIα collagen (differentiation marker of proliferating and mature chondrocytes) and type X collagen (differentiation marker of hypertrophic chondrocytes) were downregulated in the growth plates of the *Sox2-Cre-Fam20c* ^Δ/Δ^ mice (Figure 7A–7D), indicating that both the early stage differentiation and the late-stage maturation of chondrocytes were affected. BrdU labeling revealed that the absolute number of labeled proliferating chondrocytes in the *Sox2-Cre-Fam20c* ^Δ/Δ^ mice was lower (Figure 7E and 7F), but there was no statistical difference in the ratio of BrdU labeled cells to the total number of cells in the proliferative zone between the mutant mice and their WT littermates (data not shown). TUNEL assay showed that the percentage of apoptotic chondrocytes in the hypertrophic zone was reduced by ∼70% in the *Sox2-Cre-Fam20c* ^Δ/Δ^ mice (Figure 7G and 7H), which was consistent with the reports that hypophosphatemia impairs caspase-mediated apoptosis in hypertrophic chondrocytes [17], [18]. The reduction of apoptosis might be responsible for the formation of a thicker growth plate in the cKO mice.

### Inactivation of FAM20C leads to cell differentiation defects

**Figure 8:**
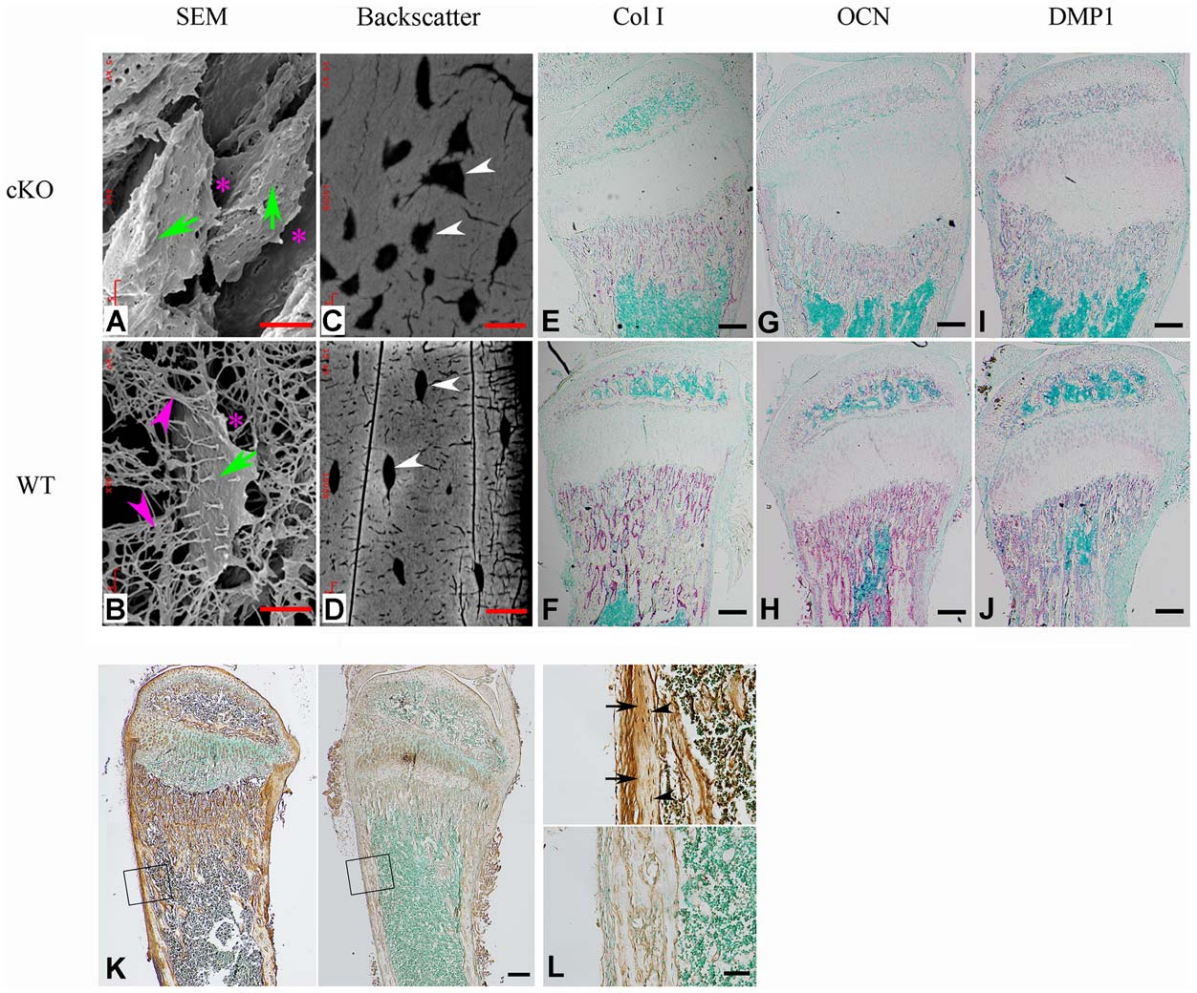
FAM20C is essential to the differentiation of bone cells. (A,B) SEM analyses of tibia from 6-week-old *Sox2-Cre-Fam20c*-cKO mice (upper) and WT littermates (lower). The osteocytes of the cKO mice showed abnormal shape (green arrows), wider periosteocytic region (red asterisk), and loss of osteocyte processes (red arrowheads), appearing immature and poorly differentiated. (C,D) Backscatter SEM analyses of the alveolar bone showed that the osteocytes in the 6-week-old *Sox2-Cre-Fam20c*-cKO mice (upper) had periosteocytic lesions (the “halo” defects, i.e., wider unmineralized regions surrounding the ostecytes) appearing as larger lacunae (arrows) compared with their WT littermates (lower). The black areas represent the unmineralized areas (osteocytes, periosteocytic region, osteocyte processes), while the grey/white areas represent the matrix that is well mineralized. (E–J) ISH staining of Col1a1, OCN, and DMP1 on the sagittal sections of tibia from 3-week-old cKO mice (upper panels) and WT littermates (lower panels), revealed significant downregulation of these terminal differentiation markers in the cKO mice. The pink/red color represents the positive ISH staining. (K) IHC staining of FGF23 in the tibia from 3-week-old cKO mice and their WT littermates revealed more FGF23 protein in the long bone of the cKO (left) than in the WT (right) mice. The brown color represents the positive IHC staining. (L) Higher magnification of the box areas in K showed more FGF23 protein in the osteocytes (arrowheads) and the bone matrices (arrows) of the cKO mice (upper) than in the WT (lower). Scale bars: 5 µm in A and B, 10 µm in C and D, 50 µm in L, 200 µm in E–K.

The osteocytes in *Fam20c* ^Δ/Δ^ mice lost normal morphology and appeared immature as shown by resin-casted scanning electron microscopy (SEM) analyses (Figure 8A and 8B), indicating a faulty maturation process from osteoblasts to osteocytes. Backscatter SEM analyses revealed periosteocytic lesions (“halo”) surrounding the osteocytes in the *Fam20c* cKO mouse bone (Figure 8C and 8D). To determine the molecular changes associated with the immaturity of osteoblasts/osteocytes, we examined their terminal differentiation markers: type Ia collagen, dentin matrix protein 1 (DMP1), and osteocalcin (OCN). ISH (Figure 8E–8J), and real-time PCR analyses (Table 2) revealed a significant downregulation of these markers in the *Sox2-Cre-Fam20c* ^Δ/Δ^ mice. Microarray analyses using total RNA extracted from the calvaria of 3-week-old *Sox2-Cre-Fam20c* ^Δ/Δ^ mice and their WT littermates indicated that among the ∼45,000 molecules evaluated, 350 genes were upregulated by over 2.0 folds and 185 were downregulated. Real-time PCR analyses on selected genes confirmed the significant changes in a number of biomineralization regulators and key players in the Wnt and TGF-β signaling pathway associated with cell differentiation (Table 2) [9]–[15], [19]–[27], suggesting an essential role of FAM20C in the differentiation and mineralization of osteogenic cells. Notably, the most striking transcriptional alteration was FGF23 (upregulated by ∼110 folds), a phosphorus regulator mainly produced by osteoblasts/osteocytes [11], [28], [29]. Immunohistochemistry against FGF23 confirmed the dramatic elevation in the bone cells and bone matrix of *Fam20c* cKO mice (Figure 8K and 8L). The transcript levels of the above genes in the *Sox2-Cre-Fam20c* ^Δ/+^ (heterozygous cKO) mice showed no difference from the WT mice (data not shown). Given the many similarities among the *Fam20c* cKO mice, *Dmp1* KO mice and Hyp mice, we examined the expression levels of *Fam20c* in the *Dmp1*- and *Phex*- deficient mice, and the levels of *Dmp1* and *Phex* in the *Fam20c* cKO mice by real-time PCR analyses. The *Fam20c* expression was not altered in the *Dmp1* KO mice and Hyp mice (data not shown). The expression of *Dmp1* was significantly downregulated (Table 2, Figure 8) while that of *Phex* was not affected (data not shown) in the *Fam20c* cKO mice. Ectonucleotide pyrophosphatase/phosphodiesterase (*Enpp1*), another molecule involved in regulating phosphorus homeostasis was slightly downregulated in the bone of the *Fam20c* cKO mice, but the change (∼1.4 folds) was not statistically significant from the WT (data not shown).

**Table 2:** Alterations of selected genes in the calvaria of 3-week-old *Fam20c*-cKO mice.

Gene symbol	Microarray (n = 1)	Q-PCR (n = 6)	Known function*
Fgf23	5.4	112±32.9	Regulator of phosphate homeostasis^9–15^
Fst	3.4	6.6±1.2	Antagonist of TGF-β/Activin pathway^19,20^
Sfrp1	3.4	1.8±0.4	Inhibitor of canonical Wnt pathway^21^
Sfrp3	3.0	1.6±0.5	Inhibitor of canonical Wnt pathway^22^
Lgr5	−3.6	−7.7±0.9	Downstream target of Wnt Pathway^23,24^
Lef1	−1.9	−3.2±0.4	Downstream target of Wnt Pathway^25^
Dmp1	−1.7	−2.3±0.6	Promoter of osteoblast maturation^11^
Ocn	NA	−9.3±0.8	Regulator of biomineralization^26^
Osterix	NA	−1.9±0.6	Promoter of osteoblast differentiation^27^

The mRNA levels in the *Fam20c*-cKO mouse calvaria were expressed as folds of change over their WT littermates.

*: For the known functions of each gene, please refer to the references in superscripts.

### The shRNA knockdown of FAM20C *in vitro* leads to similar changes in the human and mouse osteogenic cell lines

**Figure 9:**
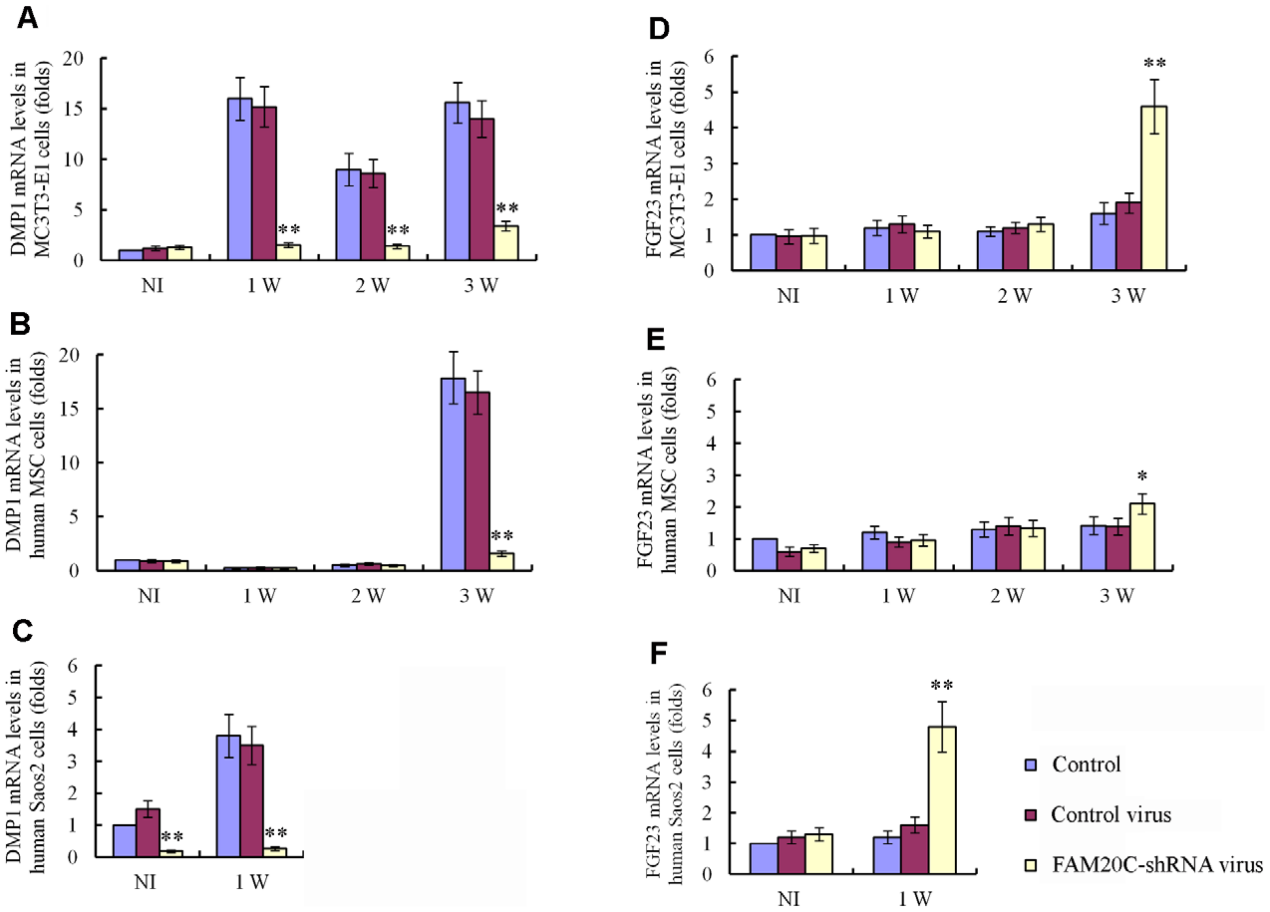
Lentiviral shRNA-mediated “knockdown” of FAM20C leads to similar alterations in the expression of DMP1 and FGF23 in human and mouse osteogenic cell lines. The cells were divided into three groups: uninfected cells, cells infected with control lentivirus expressing the scrambled shRNA and cells infected with FAM20C-shRNA lentivirus containing a mixture of 3 pieces of shRNA targeting different regions of the FAM20C mRNA. The mRNA levels of DMP1 and FGF23 in each group were determined by real-time PCR. The expression levels of the uninfected cells without osteogenic induction were taken as 1, while that of the cells infected with the control virus or FAM20C-shRNA virus were expressed as folds of change to the uninfected cells. (A) The knockdown of FAM20C led to remarkable downregulation of DMP1 in mouse MC3T3-E1 cells. Without the osteogenic induction, the expressional level of DMP1 had no significant difference among the three groups of cells. During the 3-week osteogenesis-induction process, DMP1 was significantly upregulated in the uninfected cells and cells infected with the control lentivirus, while its expression was remarkably reduced in the cells infected with FAM20C-shRNA lentivirus at 1-, 2- and 3-weeks after the start of osteogenic induction. (B) Inactivation of FAM20C led to remarkable downregulation of DMP1 in hMSC cells. The expression level of DMP1 had no significant difference among three groups in the first 2 weeks of culture in the osteogenic medium. After 3 weeks of osteogenic induction, the DMP1 expression was remarkably reduced in the cells infected with FAM20C-shRNA lentivirus when compared with the uninfected cells and cells infected with the control lentivirus. (C) Inactivation of FAM20C led to remarkable downregulation of DMP1 in Saos-2 cells. DMP1 expression was downregulated in human osteoblasts (Saos-2) infected with FAM20C-shRNA lentivirus before the osteogenic induction started, compared with the uninfected cells and cells infected with the control virus. The downregulation of DMP1 in the FAM20C-knockdown cells became more prominent after 1 week of osteogenic induction. (D) Inactivation of FAM20C led to significant upregulation of FGF23 in mouse MC3T3-E1 cells. The expression level of FGF23 had no significant difference among three groups in the first 2 weeks of osteogenic induction. After inducing the cells for osteogenic differentiation for 3 weeks, FGF23 was significantly upregulated in the cells infected with FAM20C-shRNA lentivirus, compared with the uninfected cells and cells infected with the control lentivirus. (E) Inactivation of FAM20C led to significant upregulation of FGF23 in hMSC cells. The FGF23 elevation also occurred in the hMSC cells after 3-week osteogenic induction. Note that upregulation of FGF23 in the hMSC cells was not as remarkable as in the MC3T3-E1 cells at the same time point. (F) Inactivation of FAM20C led to significant upregulation of FGF23 in Saos-2 cells. Without osteogenic induction, FGF23 level had no significant difference among the 3 groups. After 1 week of osteogenic induction, FGF23 was significantly upregulated in the cells infected with FAM20C-shRNA lentivirus, compared with the uninfected cells and cells infected with the control lentivirus. *: P<0.05; **: P<0.01; NI: without osteogenic induction; Control: uninfected control cells; Control virus: cells infected with control lentivirus; FAM20C-shRNA virus: cells infected with FAM20C-shRNA lentivirus.

The bone phenotypes in the *Fam20c* cKO mice appear opposite to those observed in the patients associated with the human *FAM20C* mutations [6]. These contradictory results raise the question of whether FAM20C functions differently between the two species. The lentiviral shRNA-mediated “knockdown” of FAM20C in mouse preosteoblasts MC3T3-E1 cells, human Saos-2 cells (osteoblasts isolated from human osteosarcoma) and human mesenchymal stem cells (hMSC) revealed a remarkable downregulation of DMP1 (Figure 9A–9C), along with a significant upregulation of FGF23 in both the human and mouse cell lines (Figure 9D–9F), indicating that FAM20C may function similarly in humans and mice.

### Recombinant FAM20C promotes cell differentiation *in vitro*

**Figure 10:**
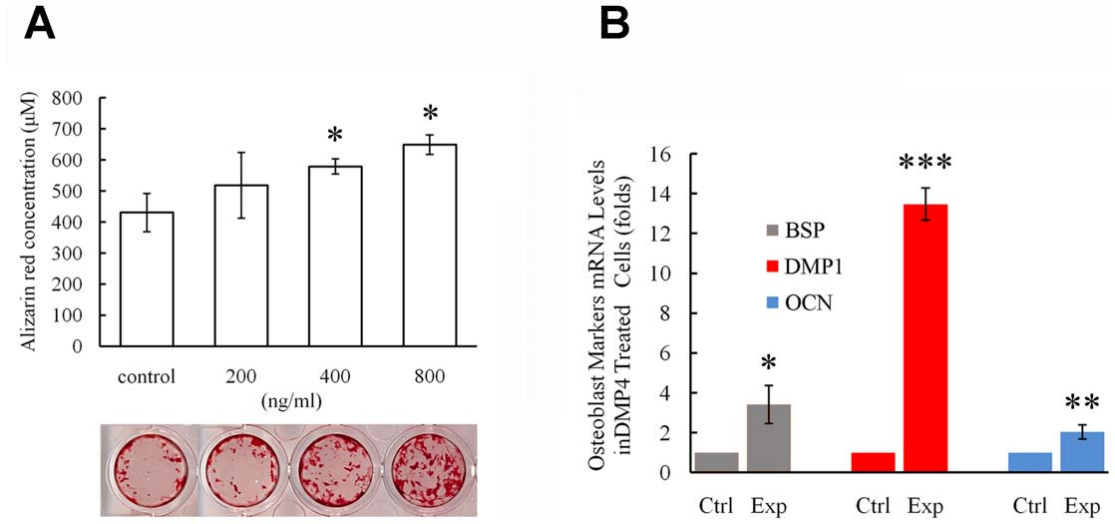
Recombinant FAM20C promotes the differentiation of MC3T3-E1 cells. (A) Alizarin red was used to stain the mineralized nodules formed by MC3T3-E1 cells treated with different concentration of recombinant FAM20C. The lower panel showed the representative culture wells from each of the experimental groups as well as the control group. The upper graph displayed the quantitative measurement of Alizarin red dye released from the mineralized nodules formed by MC3T3-E1 cells. Note that the administration of FAM20C improved the mineralized nodule formation in a dose-dependent manner. (B) Real-time PCR using RNA extracted from MC3T3-E1 cells treated with recombinant FAM20C (800 ng/ml) revealed upregulation of DMP1, OCN and BSP in the experimental groups. *: P value<0.05; **: P value<0.01; ***: P value<0.001.

To examine the role of FAM20C during osteoblast proliferation and differentiation, recombinant mouse FAM20C protein was generated by insect cells using a Bac-to-Bac baculovirus system. The recombinant FAM20C added to the culture of MC3T3-E1 preosteoblasts promoted the mineral deposition (nodule formation) in a dose-dependent manner (Figure 10A), and significantly enhanced the transcription of DMP1, osteocalcin (OCN), and bone sialoprotein (BSP) (Figure 10B). Adding recombinant FAM20C to MC3TC-E1 cells did not alter the expression of FGF23 and the proliferation rate of the cells at all tested concentrations (data not shown).

### Inactivation of FAM20C leads to hypophosphatemia and elevation of serum FGF23

Seeing the significant elevation of FGF23 in the bone cells of *Fam20c* cKO mice, we performed serum biochemistry analyses in 18-day- and 42-day-old mice (Table 3). The circulating FGF23 level was remarkably elevated in both the 18-day-old (∼200 folds) and 42-day-old (∼60 folds) cKO mice. Accordingly, the serum phosphorus level significantly decreased at both ages (∼2.5 folds in 18-day-old cKO, ∼2 folds in the 42-day-old cKO mice). The circulating PTH level was significantly elevated in both the18-day-old (∼8 folds) and the 42-day-old (∼5 folds) cKO mice. The serum 1,25(OH)_2_D_3_ level was significantly reduced (∼2 folds) in the 18-day-old cKO mice, while the serum 1,25(OH)_2_D_3_ level in the 42-day-old cKO mice was slightly higher than that of the WT, but the change in the older mice was not statistically significant. The serum calcium level slightly decreased in the 18-day- and 42-day-old cKO mice, but the reduction was not statistically significant from the WT mice. The blood urea nitrogen (BUN) level in cKO mice had no statistic difference from that of the WT mice at both ages, indicating that no renal failure was occurring in the cKO mice. The serum biochemistry results of the *Sox2-Cre-Fam20c* ^Δ/+^ (heterozygous cKO) mice were not different from their WT literates (data not shown).

**Table 3:** Serum biochemistry results in the 18-day-old and 42-day-old WT and *Fam20c*-cKO mice.

Serum biochemistry	18-day-old		42-day-old	
(mean ± SD)	WT (n = 6)	cKO (n = 6)	WT (n = 6)	cKO (n = 6)
FGF23 (pg/ml)	223.7±37.0	46771.2±11687.1**	226.3±90.8	13631.5±2585.0**
Phosphorus (mg/dl)	15.48±3.05	6.31±1.46 **	11.10±1.38	5.62±0.61**
Intact PTH (pg/ml)	132.5±114.0	1076.9±356.8**	52.3±16.8	302.6±73.1**
1,25(OH)2D3 (pmol/L)	260.3±25.1	148.3±21.9**	120.2±55.3	172.8±47.2
Calcium (mg/dl)	8.28±0.36	7.62±1.72	9.75±1.10	8.02±1.43
BUN (mg/dl)	9.1±2.3	8.6±2.7	16.1±3.6	17.2±4.3

A p-value of <0.05 was taken as statistically significant difference.

**: : significant difference from the WT mice (p<0.001). BUN: blood urea nitrogen.

The transcriptional levels of renal Klotho and NaPi-2a were slightly lower in the 18-day-old *Fam20c* cKO mice, and significantly downregulated (∼3 folds) in the 42-day-old cKO mice. The renal 1α-hydroxylase level was significantly reduced (∼2 folds) in the cKO mice at both ages. We also observed remarkable upregulation of renal 24-hydroxylase in the 18-day-old cKO mice (∼30 folds) as well as in the 42-day-old cKO mice (∼7 folds) (Table 4). The transcriptional levels of these genes in the heterozygous *Fam20c* cKO mice had no difference from their WT littermates (data not shown).

**Table 4:** Renal mRNA levels of Klotho, NaPi-2a, 1α-hydroxylase, and 24-hydroxylase in the 18-day-old and 42-day-old WT and *Fam20c*-cKO mice.

Renal mRNA levels (folds)	18-day-old		42-day-old	
(Mean ± SD)	WT (n = 6)	cKO (n = 6)	WT (n = 6)	cKO (n = 6)
Klotho	1	0.86±0.03*	1	0.28±0.03**
NaPi-2a	1	0.84±0.03*	1	0.35±0.05**
1α-hydroxylase	1	0.53±0.03**	1	0.63±0.07**
24-hydroxylase	1	30.33±1.83**	1	6.87±0.77**

The renal mRNA level of WT revealed by real-time PCR was taken as 1, while that of the FAM20C-deficient kidney was expressed as folds of change to the WT.

*: : significant difference from WT mice (p<0.05).

**: : significant difference from WT mice (p<0.001).

### Expression of the *Fam20c* transgene rescued the defects of *Sox2-Cre-Fam20c* ^Δ/Δ^ mice

**Figure 11:**
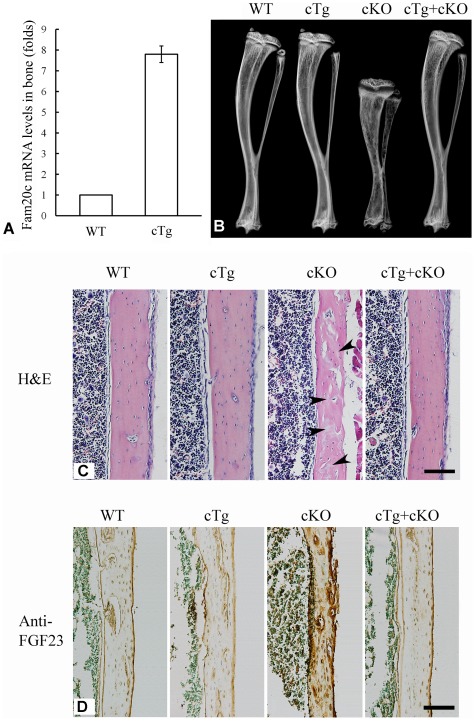
Expression of the *Fam20c* transgene rescued the defects of *Sox2-Cre-Fam20c*-cKO mice. (A) The expression level of the *Fam20c* transgene was evaluated by real-time PCR using mRNA extracted from the long bones of 6-week-old mice. One line of the transgenic mice (cTg) with the highest expression level of the *Fam20c* transgene (∼8 folds over that of their WT littermates) was used to rescue the defects in the cKO mice. (B) Plain X-ray analyses of tibias from 6-week-old mice. The bone of mice expressing the *Fam20c* transgene in the wild type background (cTg) had no difference from the WT. Note the tibia of the cKO mice expressing the *Fam20c* transgene (cTg+cKO) had no difference from the WT, indicating that the expression of the transgene fully rescued the bone defects of *Sox2-Cre-Fam20c*-cKO mice (cKO). (C) H&E staining on the sagittal sections of tibias from 6-week-old WT, cTg, cKO and cTg+cKO mice. The tibia cortical bone of the cKO mice had more osteoids (grey areas indicated by arrowheads) and thinner cortical bone, while the cortical bones of the WT, cTg and cTg+cKO mice showed normal structures. These findings further demonstrate that expressing the *Fam20c* transgene completely rescued the bone defects of the *Fam20c* cKO mice. (D) Anti-FGF23 IHC staining on the sagittal sections of tibias in the 3-week-old WT, cTg, cKO and cTg+cKO mice. The cortical bone of tibias in the WT, cTg and cTg+cKO mice showed very similar level of FGF23 expression while that of the cKO mice demonstrated a significantly elevated FGF23 expression. These results indicate that expressing the *Fam20c* transgene rescued the altered FGF23 expression in the cKO mouse bone. Scale bars: 100 µm in C and D.

Conditional transgenic (cTg) mice expressing the full length FAM20C were generated to test the gain of function *in vivo*. We obtained 15 lines of cTg mice, and three of them with the transgene expression levels of 4∼8 folds over those of the WT littermates were further analyzed. One line with the highest expression level of the transgene (approximately 8 folds over the WT, Figure 11A) was characterized in detail. No abnormalities were observed in the bone of any of the cTg mice by postnatal 6 weeks (Figure 11B). In addition, by breeding the cTg mice with the *Fam20c* cKO mice, we obtained mice expressing the transgene in the *Fam20c* knockout background (designated “*Sox2-Cre-Fam20c* ^Δ/Δ^-*cTg* mice”). The *Sox2-Cre-Fam20c* ^Δ/Δ^-*cTg* mice had no abnormalities in the skeleton by postnatal six weeks (Figure 11B and 11C), indicating that expressing the transgene fully rescued the defects of the *Fam20c*-deficient mice. Additionally, IHC staining using the anti-FGF23 antibodies showed that the expression of the *Fam20c* transgene rescued the altered expression of FGF23 in the *Fam20c*-deficient bone (Figure 11D). The fact that overexpressing FAM20C in the WT background did not cause defects in the bone, along with the observation that overexpressing the transgene rescued the *Fam20c*-deficient abnormalities, has provided further evidence that the defects in the hard tissues of the *Sox2-Cre-Fam20c* ^Δ/Δ^ mice resulted from the loss-of-function, and were not due to the gain-of-function.

In summary, the multipronged approaches in this study demonstrated that inactivation of FAM20C in mice led to rickets/osteomalacia, along with altered levels of serum phosphate and FGF23. The manifestations of the *Fam20c*-deficient mice are consistent with a diagnosis of hypophosphatemic rickets. The *Fam20c*-deficient cells in the mineralized tissues appeared immature and incapable of forming healthy tissues. It is likely that a combination of cell differentiation failure and hypophosphatemia resulting from the FGF23 excess led to the skeletal defects in the *Fam20c*-deficient mice.

## Discussion

Little is known about FAM20C, a new molecule. *In vitro* studies have shown that it promotes the differentiation and mineralization processes of undifferentiated mesenchymal cells and odontoblasts [5], whereas human genetic studies suggested that FAM20C might be a down-regulator (inhibitor) of bone formation and/or mineralization [6]. To answer critical questions regarding the biological roles of FAM20C, we generated *Fam20c* conditional knockout mice, in which exons 6–9 (majority of the conserved CCD region) were ablated. It is worth noting that most of the mutations identified in the patients with lethal osteosclerotic bone dysplasia were in exons 6–9 [6]. The *Fam20c* conditional knockout mice developed hypophosphatemic rickets but not osteosclerosis. We believe that the abnormalities in the *Fam20c* cKO mice resulted from the loss-of-function and were not due to the gain-of-function for this protein. This belief is based on the following observations: 1) deleting exons 6–9 (majority of the CCD) in the *Fam20c* cKO mice was most likely to inactivate this molecule; 2) the inheritance of the phenotypic changes in *Fam20c* cKO mice occurred in an autosomal recessive trait, while the gain-of-function is usually inherited in an autosomal dominant manner; 3) transgenic mice overexpressing the *Fam20c* transgene were normal; 4) the overexpression of the *Fam20c* transgene fully rescued the phenotypic changes in the *Fam20c* cKO mice; and 5) recombinant FAM20C promoted the differentiation and mineralization of MC3T3-E1 cells in a dose-dependent manner. These data combined with a significant downregulation of osteoblast differentiation markers in cKO mice suggest that FAM20C is essential to the differentiation of mineralizing cells and promotes the formation and mineralization of hard tissues, and thus, inactivation of this molecule leads to differentiation failure of the cells forming these tissues. Additionally, the *Fam20c* cKO mice developed hypophosphatemia with a remarkable elevation of the serum FGF23 level. We believe that a combination of cell differentiation failure and hypophosphatemia caused by the increase of serum FGF23 led to the skeletal defects in the *Fam20c* conditional knockout mice.

In 2007, Simpson et al. reported that the mutations of human *FAM20C* are associated with an osteosclerotic phenotype in some patients [6]. In a later study by the same group [8], Simpson et al. identified *FAM20C* mutations in two patients whom they believed were suffering from a “different type of Raine Syndrome”; these two patients did not show a generalized increase in bone density, with one case showed “manifestations consistent with a diagnosis of hypophosphatemic rickets”, as the authors stated. The osteosclerotic phenotype in some patients with *FAM20C* mutations appears opposite to that observed in the *Fam20c*-deficient mice. These contradictory results raise questions of whether different domains/fragments of FAM20C protein have different functions or if their functions are different between humans and mice.

In previous reports, the human *FAM20C* mutations in the osteosclerotic patients include point missense mutations and “splicing” mutations [6], [8]. The point mutations were located in different regions of the gene, including the region encoding the N-terminal portion of the protein and that corresponding to the C-terminal part. The 1309G→A mutation (D437N, in exon 7) observed in the “hypophosphatemic rickets”-like patient (Case 1 in [8]) is located between the two missense mutations 1121T→G (L374R, in exon 6) and 1603C→T (R535W, in exon 10) and was very close to a splicing mutation C1322-2A→G (in intron 7). The latter three mutations initially reported by Simpson et al. were associated with a generalized hypermineralization in the patients [6], while the former one was associated with “hypophosphatemic rickets” [8]. The secreted form of mouse FAM20C contains 553 amino acid residues (excluding a putative 26-amino acid signal peptide), and its calculated molecular mass is approximately 63 kDa. In our previous study [7], Western immunoblotting analyses of the culture medium from HEK-293 cells transfected with a pMES construct containing full-length mouse FAM20C cDNA demonstrated a single protein band at approximately 65 kDa, consistent with the expected mass of full-length mouse FAM20C. We did not observe any lower molecular weight protein bands that could be recognized by the anti-FAM20C antibodies. Similar results were documented in the analyses of the mouse C3H10T1/2 cells and MC3T3-E1 cells transfected with the FLAG-tagged FAM20C [5]. These observations indicate that mouse FAM20C may not be proteolytically processed into fragments. Taken together, these human and mouse data do not support the contention that different domains or fragments of FAM20C may perform different functions.

In this study, the lentiviral shRNA-mediated knockdown of FAM20C in the human mesenchymal stem cells and human osteoblasts led to a remarkable downregulation of DMP1, along with a significant upregulation of FGF23 (Figure 9B, 9C, 9E, and 9F). The findings in the human cells are consistent with the results in the shRNA-knockdown of FAM20C in mouse MC3T3-E1 cells (Figure 9A and 9D) and with the observations in the *Fam20c* conditional knockout bone (Figure 8, Table 2); these results indicate that FAM20C is likely to function similarly in humans and mice. Clearly, more studies are warranted to further clarify the discrepancy between the human and mouse data.

**Figure 12:**
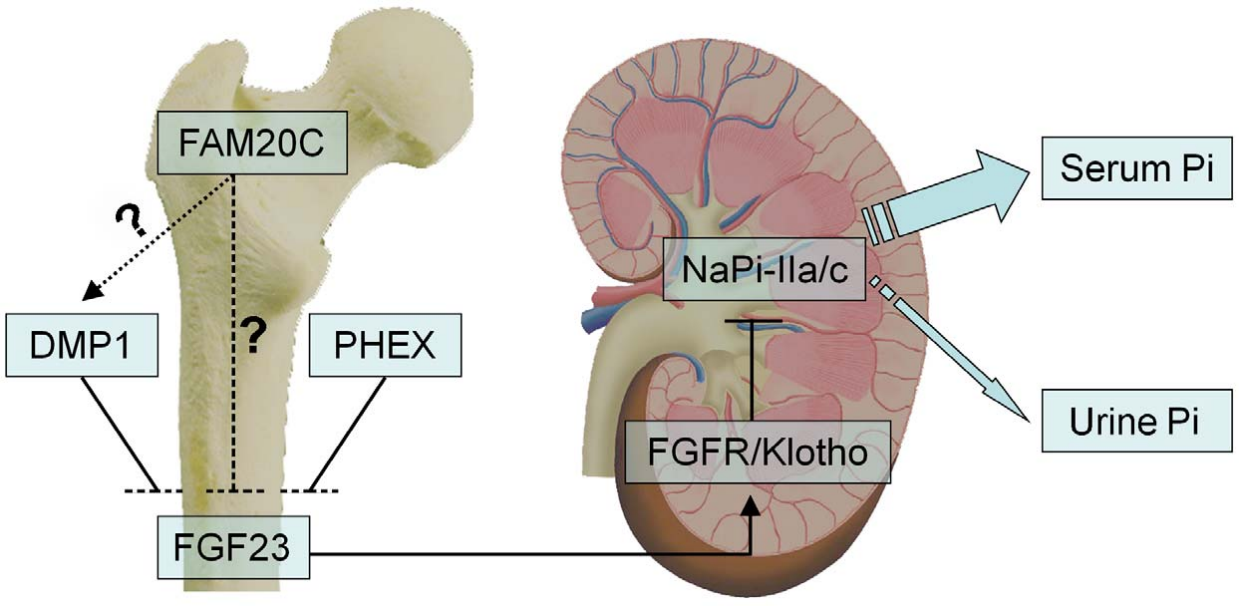
FAM20C may mediate phosphate homeostasis via FGF23. FAM20C, DMP1 and PHEX may share similar mechanisms in their involvement in the bone-kidney axis when regulating phosphate homeostasis via FGF23. FAM20C secreted by osteoblasts/osteocytes may regulate the phosphaturic hormone FGF23 expression in these cells. FGF23 targets the Klotho/FGF receptor (FGFR) complexes in the kidney, reducing the expression of the renal sodium-phosphate cotransporters NaPi-2a/2c in the proximal tubules, thereby accelerates phosphate excretion into the urine and helps maintaining the serum phosphate levels in the normal range. While inactivation of FAM20C led to remarkable reduction of DMP1 in the mice and osteogenic cell lines, and recombinant FAM20C significantly increased the expression of DMP1 in MC3T3-E1 cells, it is unclear if FAM20C regulates FGF23 and phosphate homeostasis *via* DMP1 (dotted arrow) or FAM20C directly regulates FGF23 (dashed line).

As a growth factor, FGF23 principally functions as a phosphaturic hormone via binding to the Klotho/FGF receptor (FGFR) complexes in the kidney [30], [31]. The binding of FGF23 to FGFR accelerates phosphate excretion into the urine, thereby inducing a negative phosphate balance, which helps maintain the serum phosphate levels in the normal range under physiological conditions (Figure 12). Elevation of the FGF23 plasma level is known to lead to renal phosphate-wasting and hypophosphatemia [11]–[15], [28], [32]–[35]. The main sources of FGF23 are the osteoblasts and osteocytes in the skeleton [11], [28], [29], and a number of studies have shown that inactivating mutations in certain molecules expressed by these bone cells increase the plasma level of FGF23, which leads to hereditary hypophosphatemic rickets [10], [11], [13], [14]. Inactivating mutations in the phosphate-regulating gene with homologies to endopeptidases on the X chromosome (*PHEX*) cause X-linked hypophosphatemic rickets (OMIM 307800) [10], and loss of *DMP1* activity results in autosomal-recessive hypophosphatemic rickets (OMIM 241520) [11], [13]. The phenotypic changes in the *Fam20c*-conditional knockout mice share many similarities (hypomineralization, elevation of FGF23, hypophosphatemia) with those observed in the *PHEX*- and *DMP1*-deficient subjects. As in the cases of *PHEX*- and *DMP1*-deficiency, FGF23 was overexpressed in the bones of the *Fam20c* cKO mice (Figure 8K and 8L, Table 2). The overproduction of FGF23 by the bone cells is likely to be responsible for the elevation of this protein in the serum.

Mutations in four genes, *FGF23* itself, *PHEX*, *DMP1* and *ENPP1*, have been reported to remarkably increase the plasma levels of FGF23, leading to hereditary hypophosphatemic rickets [9]–[11], [13]–[15]. *Dmp1*-, *Phex*- and *Fam20c*-deficient mice shared similarities in osteomalacia, hypophosphatemia and the remarkable elevation of FGF23 in the circulation and skeleton. Interestingly, an alteration of *Fam20c* expression was not observed in the *Dmp1* KO mice or Hyp mice, while remarkable downregulation of *Dmp1* (but not *Phex*) was observed in the *Fam20c* cKO mice. An up-regulation of *Dmp1* was observed in MC3T3-E1 cells treated with recombinant FAM20C. On the other hand, a remarkable down-regulation of *Dmp1* was seen in human and mouse osteogenic cell lines treated with FAM20C-shRNA. These findings, along with the similarities of skeletal and serum changes between the *Fam20c*-deficient and *Dmp1*-deficient mice, raise the question of whether FAM20C regulates DMP1 (Figure 12). Clearly, further studies are warranted to answer this question. Additionally, there are still a pool of patients with hereditary hypophosphatemic rickets whose etiology is unknown [35], [36], and our discovery that the inactivation of *Fam20c* in mice results in hypophosphatemic rickets necessitates a consideration of screening *FAM20C* in such patients.

High level FGF23 reduces the expression of renal vitamin D 1α-hydroxylase and increases the expression of the catabolic 25-hydroxyvitamin D 24-hydroxylase, thus leading to decreased levels of 1,25(OH)_2_D_3_ in the serum [15], [37], [38]. In the 18-day-old *Fam20c* cKO mice, the 1,25(OH)_2_D_3_ level was significantly lower, while in the 42-day-old cKO mice the 1,25(OH)_2_D_3_ level managed to return to the normal range (or a not significantly higher level). Similar shifts in the serum 1,25(OH)_2_D_3_ level with aging have been observed in other hypophosphatemic models such as the *Fgf23* transgenic mice, Hyp mice and *Dmp1*-KO mice that have high levels of circulating FGF23 [11], [12], [39].

While elevation of serum FGF23 reduces the expression of the renal 1α-hydroxylase, hypophosphatemia is normally a stimulator for renal 1*α*-hydroxylase expression to increase circulating 1,25(OH)_2_D_3_ [40]; the stimulating effect of hypophosphatemia on the 1α-hydroxylase expression has been well illustrated in the NaPi2a knockout mice (with lower phosphate and lower FGF23 levels in the serum), in which the serum 1,25(OH)_2_D_3_ level is elevated due to the increased 1*α*-hydroxylase expression stimulated by hypophosphatemia [41]. The *Fam20c*-cKO mice displayed a decreased level of renal 1*α*-hydroxylase in the presence of hypophosphatemia, indicating that in these mutant mice, the negative modulation of FGF23 on the expression of the 1*α*-hydroxylase may outweigh the stimulating effect of hypophosphatemia on the 1*α*-hydroxylase expression.

In comparison with the *Fgf23* transgenic mice, *Dmp1*-KO mice and Hyp mice, a more remarkable upregulation of 24-hydroxylase was observed in the kidney of the *Fam20c*-cKO mice, which may be due to the fact that *Fam20c*-cKO mice have a higher serum FGF23 level than the former three [11], [12], [33]. A higher level of 24-hydroxylase in the 18-day-old cKO mice than that in the 42-day-old *Fam20c* cKO mice (∼30-fold versus ∼7-fold elevation) may be responsible for the significantly lower serum 1,25(OH)_2_D_3_ level in the younger animals. In the older *Fam20c* cKO mice, a significant reduction of FGF23 co-receptor Klotho, along with a relatively lower serum FGF23 level than that in the younger cKO mice, may attenuate the FGF23-elevation effects on circulating 1,25(OH)_2_D_3_ and thus may help maintain a relatively normal serum 1,25(OH)_2_D_3_ level in the older cKO mice. More likely, the lower serum level of 1,25(OH)_2_D_3_ in the younger *Fam20c* KO mice may have triggered the overproduction of PTH, as in the cases of *Fgf23* transgenic mice and Hyp mice. The secondary hyperparathyroidism may play a critical role for reversing the 1,25(OH)_2_D_3_ level to the normal range in the older *Fam20c* cKO mice [42], [43]. In addition, the elevated PTH may synergize with the high level of serum FGF23 to increase renal phosphate excretion by reducing the expression of NaPi2a in the proximal tubules [38], [44]; a significant reduction of NaPi2a was observed in the kidney of the *Fam20c* cKO mice. In the end, 1,25(OH)_2_D_3_ may maintain a relatively normal level in the older *Fam20c* cKO mice at the expense of a significant phosphorus wasting.

The skeletal defects of the *PHEX*- and *DMP1*-deficient subjects are believed to be due to the combined effects of two factors: 1) the intrinsic defects of the *PHEX*- and *DMP1*-deficient cells that prevent them from forming and mineralizing ECM properly and 2) hypophosphatemia [11], [33], [45], [46]. The *Fam20c*-deficient cells responsible for forming the mineralized tissues appeared immature and showed altered expression levels for molecules associated with cell differentiation. While hypophosphatemia in the *Fam20c* conditional knockout mice can be attributed to the overproduction of FGF23 in the abnormal skeleton, the direct cause of cell differentiation failure may be complicated. As stated above, the defects in the mineralized tissues of the *Fam20c* conditional knockout mice could be the combined results of cell differentiation failure and hypophosphatemia. Although the way FAM20C regulates cell differentiation has not yet been defined in this study, our data suggest that FAM20C may regulate the differentiation and function of the mineralizing cells by participating in certain signaling pathways.

Several lines of evidence suggested that FAM20C might be associated with the canonical Wnt signaling pathway. The Wnt canonical pathway inhibitors, secreted frizzled related protein 1 (Sfrp1) and Sfrp3, were upregulated in the *Fam20c*-deficient mice. Accordingly, the downstream target genes of Wnt pathway, leucine-rich repeat-containing G protein-coupled receptor 5 (Lgr5) and lymphoid enhancer-binding factor 1 (Lef1) were significantly downregulated in the *Fam20c*-deficient bone. Lgr5 is a stem cell marker and a Wnt pathway regulator which has been identified in multiple tissues including bone marrow cells [23], [24]. Lef1 is a Wnt-responsive transcription factor that associates with β-catenin and has been documented to increase osteoblast activity and trabecular bone mass [25]. However, not all of the findings support the postulation that FAM20C is a participant in the Wnt signaling pathway. For example, the level of axis inhibition protein 2 (Axin2), a putative Wnt downstream target gene, was unchanged in the *Fam20c*-deficient bone. In addition to the molecules in the Wnt signaling pathway, Follistatin (Fst), a potent inhibitor of Activin and the TGF-β pathway, was significantly upregulated in the bones of 3-week and 6-week-old *Fam20c*-deficient mice. Fst has been reported to inhibit ameloblast and osteoblast differentiation [19], [20], suggesting a possible association between FAM20C and the TGF-β pathway.

Type II and Type X collagen were downregulated in the growth plates of the *Fam20c* conditional knockout mice. The downregulation of these genes may arise from the intrinsic defects of chondrocytes or occur as a systematic consequence of hypophosphatemia. It has been well documented that hypophosphatemia significantly decreases programmed cell death in growth plates by impairing caspase-mediated apoptosis of hypertrophic chondrocytes [17], [18].

In conclusion, the results in this investigation have demonstrated the crucial role of FAM20C in osteogenesis. Our findings indicate that FAM20C is essential to the differentiation of osteoblasts/osteocytes and is involved in the regulation of phosphate homeostasis via the mediation of FGF23.

## Methods

### Ethics statement

All animal procedures were performed in accordance with the NIH *Guide for the Care and Use of Laboratory Animals* and approved by the Institutional Animal Care and Use Committee of Texas A&M Health Science Center, Baylor College of Dentistry (Dallas, TX, USA).

### Generation of *Fam20c* conditional knockout mice lacking exons 6∼9

To generate the *Fam20c* conditional knockout mice, a 2.2 kb targeting fragment spanning exons 6∼9 of mouse *Fam20c* was produced by PCR using the genomic DNA of WW6 ES cells as a template (forward primer: 5′-CTCTCGGGTGAGGCTGTAAG-3′; reverse primer: 5′-AGATCTCTTAGGGAAGAGGGGTCAGG-3′). The fragment was subcloned into a floxed BamHI site upstream of an Frt flanked mcl-Neo cassette in the conditional targeting vector pFlox-Frt-Neo [47]. A 2.5 kb 5′ homologous arm was generated by PCR (forward primer: 5′-CTCGAGTGGGTGTGTCAGGAATCGTA-3′; reverse primer: 5′-CTCGAGACCCGAGAGCAACCACATAC-3′), and subcloned into the XhoI site of the targeting vector. A 4.2 kb 3′ homologous arm with EagI sites at both ends was generated by PCR (forward: 5′-TCGGCCGTTGGACATAGGCTCCCAAAG-3′; reverse: 5′-TGTGCAGGATTGAGAACCAG-3′), and subcloned into the NotI site of the targeting vector. Finally, a negative selection PGK-DTA (diphtheria toxin A) cassette was subcloned into the NotI site downstream of the 3′ homologous arm in the targeting vector (Figure 1A). The final targeting construct was linearized with SacII and electroporated into W4 ES cells (Transgenic Mouse Core Facilities, University at Albany, Rensselaer, NY, USA). Clones were picked after G418 selection. Genomic DNA was extracted from the ES cells in duplicate plates, and PCR analyses were performed to screen the targeted clones (5′ screen primers: 5′S-F: 5′-TTTCTGTCCTAGGTAAGGGTGAAG-3′, 5′S-R: 5′-ACTGCTCGATGAAGTTCCTATTCT-3′; 3′ screen primers: 3′S-F: 5′-TGTTCGGATCGAAGTTCCTATACT-3′, 3′S-R: 5′-ACAGCTTCTTGAATTGGGATAAAG-3′) (Figure 1A and 1B). The 5′ and 3′ screening PCR products were sequenced to confirm the correct targeting and the presence of 5′ and 3′ loxP sites.

Two correctly targeted ES cell clones (Clone 286 and 297, Figure 1B) were identified. The neo cassette was removed from the targeted ES clones by transient transfection of pCAGGS-flpE-puro vector (Addgene plasmid 20733) [48]. The removal of the neo cassette was confirmed by PCR (forward: 5′-GCATCTGCAGACCGAGCCCA-3′, reverse: 5′-CCCCCTGTCCTGAGGGCTGA-3′). Random integration of pCAGGS-flpE-puro DNA into ES cell genome was excluded by PCR analyses (forward: 5′-GCATGGCCGAGTTGAGCGGT-3′, reverse: 5′-GGTGACGGTGAAGCCGAGCC-3′). The targeted ES clones recovered from the master plates were injected into the blastocysts of C57BL/6 mice in the Transgenic Core Facility of the University of Texas Southwestern Medical Center at Dallas. Male chimeras were crossbred with C57BL/6 females to produce F1 agouti offspring. The floxed alleles of F1 agouti mice were genotyped by PCR analyses (see below). To generate *Fam20c* conditional knockout mice, the F1 heterozygous mice (*Fam20c* ^flox/+^) were first crossbred with Sox2 promoter-driven Cre transgenic mice (Jackson Laboratory) that express the Cre recombinase transgene in the epiblasts at E6.5; this breeding gave rise to *Sox2-Cre-Fam20c* ^Δ/+^ mice in which exons 6∼9 were removed from one allele of the *Fam20c* gene. The *Sox2-Cre-Fam20c* ^Δ/+^ mice were inbred to produce *Fam20c* conditional knockout (cKO) mice designated as “*Sox2-Cre-Fam20c* ^Δ/Δ^ mice”, in which exons 6∼9 were removed from both alleles of the *Fam20c* gene.

Genotypes were determined by PCR analyses using genomic DNA extracted from the mouse tails. The floxed allele was distinguished from the wild type (WT) allele by PCR analyses using a mixture of three primers (a: 5′-TCCAGCTTGCTAGGGCTCTGACC-3′, b: 5′-CTATGTCCAACGGCCGCAGCTT-3′, and c: 5′-GTCCTGAGGGCTGACCCAAGACTA-3′) (Figure 1A and 1C). The null allele (i.e., that with exons 6∼9 removed) arising from Cre-loxP recombination events was detected by PCR using primers Rec-F: 5′-GTGGTCTCTGCCGCTGATGTACC-3′ and Rec-R: 5′-TTTGGGAGCCTATGTCCAACGGCC-3′ (Figure 1A and 1C). Genotyping for the Cre transgene was determined by PCR using primers Cre-F: 5′-CCCGCAGAACCTGAAGATG-3′ and Cre-R: 5′-GACCCGGCAAAACAGGTAG-3′.

We also used the 3.6 kb Col 1a1 promoter-Cre transgenic mice (The Jackson Laboratory) to generate “*Col1a1-Cre-Fam20c* ^Δ/Δ^ mice”, in which FAM20C was inactivated in tissues expressing type I collagen. We first crossbred the *Fam20c* ^flox/+^ mice with 3.6 kb Col 1a1 -Cre mice, and then inbred the offspring of the Col1a1-Cre-*Fam20c* ^Δ/+^ mice to get *Col1a1-Cre-Fam20c* ^Δ/Δ^ mice.

### Generation of *Fam20c* conditional transgenic mice and mice expressing the transgene in the *Fam20c* conditional knockout background

To generate *Fam20c* conditional transgenic mice, the coding sequence of mouse *Fam20c* cDNA was subcloned into a bicistronic pMES vector [49] downstream to a chicken β-actin promoter and upstream to an IRES-EGFP cassette as previously described [7]. A floxed STOP cassette was inserted between the β-actin promoter and the *Fam20c* sequence to block the transcription of the transgene. The *Fam20c* transgene can be activated only after the floxed STOP cassette is removed by Cre recombinase [7]. This *Fam20c* conditional transgenic construct was linearized and injected into the pronuclei of fertilized eggs from the C57BL/6 mice in the Transgenic Core Facility of the University of Texas Southwestern Medical Center at Dallas. Fifteen lines of transgenic mice with the conditional *Fam20c* transgene were identified by PCR genotyping using primers located in the exogenous EGFP sequences (GFP-forward: 5′-ACGTAAACGGCCACAAGTTC-3′ and GFP reverse: 5′-TGCTCAGGTAGTGGTTGTCG -3′). The mice carrying the conditional transgene were crossbred with the Sox2-Cre transgenic mice to remove the floxed STOP cassette between the chicken β-actin promoter and the *Fam20c* cDNA, thereby allowing the *Fam20c* transgene to be transcribed. These conditional transgenic mice (cTg mice) were genotyped using the aforementioned GFP primers and Cre primers. The expression level of the *Fam20c* transgene in the long bones of each line was evaluated by quantitative real-time PCR. Three lines with the transgene expression levels of 4∼8 folds over that of the WT mice were further analyzed.

To generate mice expressing the transgene in the *Fam20c* conditional knockout background, we crossbred *Sox2-Cre-Fam20c* ^Δ/+^ mice with cTg mice expressing the highest level of the trangene to obtain *Sox2-Cre–Fam20c* ^Δ/+^-*cTg* mice, which were then inbred to produce mice expressing the transgene in the *Fam20c* conditional knockout background (designated “*Sox2-Cre-Fam20c* ^Δ/Δ^-*cTg* mice”). PCR analyses with primers used in the identification of the *Fam20c* conditional knockout mice and cTg mice were employed in the genotyping of the *Sox2-Cre-Fam20c* ^Δ/Δ^-*cTg* mice.

All mice in this study were fed with Teklad 6% fat mouse/rat diet (Harlan, IN) and some of the chow contents are as follows: Calcium 2.4%, phosphorus 1.5%, vitamin D 3.0 IU/g.

### RT–PCR

Femurs from the *Fam20c* conditional knockout mice, *Sox2-Cre-Fam20c* ^Δ/Δ^-*cTg* mice and the WT littermates were dissected, and total RNA was extracted using an Rneasy Mini Kit (Qiagen) according to the manufacturer's instructions. The total RNAs were converted into cDNAs using a Reverse Transcription Kit (Qiagen). RT-PCR was performed to examine the lack of *Fam20c* mRNA in the cKO mice using two sets of primers: Set 1-F: 5′-TGCGGAGATCGCTGCCTTCC-3′, Set 1-R: 5′-GCCACTGTCGTAGGGTGGCG-3′; Set 2-F: 5′-GAGAGCAGGAGACGCCGCCT-3′, and Set 2-R: 5′-CCACCACACTGCTCAGCCCG -3′ (Figure 2A).

### Alizarin Red/Alcian Blue staining of the skeleton

One-week-old *Sox2-Cre-Fam20c* ^Δ/Δ^ mice and WT littermates were skinned, eviscerated and fixed in 95% ethanol. Alizarin Red/Alcian Blue staining of the skeletons was performed to visualize the skeleton and the overall mineralization levels, as described previously [50].

### X-ray radiography

The narcotized mice or the dissected jaws and long bones from hind legs were analyzed using X-ray radiography (Faxitron MX-20DC12). Micro-computed tomography (Micro-CT) analyses were performed using a Scanco micro-CT35 imaging system (Scanco Medical) with a medium-resolution scan (7.0 µm slice increment) on the dissected tissues, as previously reported [51]. The images were reconstructed with the EVS Beam software using a global threshold at 240 Hounsfield units.

### Preparation of decalcified sections and H&E staining

Tibia and jaw tissues dissected from the mice were fixed with 4% paraformaldehyde in 0.1% diethyl pyrocarbonate (DEPC)-treated PBS solution at 4°C overnight and then were decalcified in 0.1% DEPC-treated 15% EDTA (pH 7.4) at 4°C for 8 days. The tissues were processed for paraffin embedding, and serial 5 µm sections were prepared for histological analyses. H&E staining was performed as previously described [17].

### Cell proliferation and TUNEL assays

BrdU was administrated to 3-week-old *Sox2-Cre-Fam20c* ^Δ/Δ^ mice and WT littermates at a dosage of 1 ml per 100 g body weight by intraperitoneal (i.p.) injection according to the manufacturer's instructions (Invitrogen). Two hr after the injection, the mice were sacrificed. Tibias were dissected and processed for paraffin embedding, and 5 µm sections were prepared for BrdU detection using a Zymed BrdU staining kit (Invitrogen) following the manufacturer's instructions. Apoptosis in growth plates was examined by TUNEL assay using the ApopTag Plus Fluorescein *In Situ* Apoptosis Detection Kit (Millipore) according to the manufacturer's instructions. Six serial sections from each of six individual samples of *Sox2-Cre-Fam20c* ^Δ/Δ^ mice and WT littermates were counted, and the data were analyzed statistically.

### Immunohistochemistry (IHC) staining

The IHC experiments were carried out using an ABC kit and a DAB kit (Vector Laboratories) according to the manufacturer's instructions. A polyclonal C-terminal anti-FAM20C antibody was used at a concentration of 1 µg IgG/ml for the IHC experiments, as previously described [7]. A monoclonal FGF23 antibody (Cell Essentials) was used at a dilution of 1∶400 following the manufacturer's instruction. A polyclonal biglycan antibody (LF-159) was kindly provided by Dr. Larry Fisher (NIDCR, National Institutes of Health) [52]. Methyl green was used for counterstaining.

### *In situ* hybridization (ISH)

A 380 bp fragment from the region of exons 6–9 of *Fam20c* cDNA was obtained by PCR using forward primer 5′- CCGAGCATGCCCTGTGTGGG -3′ and reverse primer 5′- TGCAGCACTGATGAAGAGGAGCG -3′. The PCR product was subcloned into the pCRII-TOPO vector (Invitrogen) and then linearized with EcoRV to synthesize the antisense RNA probes using the Sp6 RNA polymerase or with HindIII to synthesize the sense RNA probes using the T7 RNA polymerase. The constructs used to generate RNA probes for DMP1, osteocalcin, collagen type I, collagen type II, and collagen type X were provided by the laboratory of Dr. Jian Q. Feng. The constructs were linearized and labeled with digoxigenin (DIG) using a RNA Labeling Kit (Roche, Indianapolis, IN) as previously described [7]. DIG-labeled RNA probes were detected by an enzyme-linked immunoassay with a specific anti-DIG-AP antibody conjugate (Roche) and an improved substrate (Vector Laboratories), which produces a red color for positive signals, according to the manufacturer's instructions. Methyl green was used for counterstaining.

### Preparation of undecalcified histology sections

Tibias dissected from 6-week-old mice were fixed in 4% paraformaldehyde overnight. The specimens were dehydrated through a graded series of ethanol (70–100%) and embedded in methylmethacrylate (MMA) without prior decalcification, as previously described [53]. Ten µm sections were prepared for Goldner staining and double-labeling fluorescent analysis.

### Double fluorochrome labeling

Double fluorescence labeling was performed as previously described [54]. Briefly, calcein (5 mg/kg i.p.; Sigma-Aldrich) was administered to the 5-week-old mice, followed by injection of an Alizarin Red label (20 mg/kg i.p.; Sigma-Aldrich) 7 days later. The mice were sacrificed 48 hr after the injection of the second label and the tibias were embedded in MMA; 10 µm sections were then prepared. The unstained sections were viewed under epifluorescent illumination using a Nikon E800 microscope, interfaced with Osteomeasure histomorphometry software (version 4.1, Atlanta, GA). The mean distance between the two fluorescent labels was determined and divided by the number of days between labels to calculate the mineral deposition rate (µm/day).

### Goldner's Masson Trichrome staining

Ten µm undecalcified sagittal sections from the tibias were stained using Goldner-Masson trichrome assay, as previously described [55]. The cortical bone areas in the midshaft were photographed using a Nikon microscope at 10× with Bioquant OSTEO v.7.20.10 (R&M Biometrics) software. Unmineralized osteoid stains red, and mineralized bone stains green/blue.

### Resin-casted scanning electron microscopy (SEM) and backscattered SEM

For resin-casted osteocyte lacunocanalicular SEM, the surface of the MMA embedded tibia was polished, acid-etched with 37% phosphoric acid for 2–10 s, washed with 5% sodium hypochlorite for 5 min and then coated with gold and palladium and examined by FEI/Philips XL30 Field emission environmental SEM. Backscattered SEM was performed as we previously described [56]

### Quantitative real-time PCR and microarray analyses

Total RNAs were isolated from the calvaria bones and decapsulated kidneys of 3-week-old mice and cultured cells. The kits for RNA extraction and reverse transcription were the same as in the RT-PCR experiments. Quantitative real-time PCR was performed on a Bio-Rad CFX96 system (Bio-Rad) using SYBR Green Master Mix (Stratagene). The Ct values were normalized to the reference gene 18s rRNA (SABiosciences), and then expressed as fold changes compared with the experimental controls. The primers for human 18s rRNA, mouse 18s rRNA, mouse *Fam20c* (NM_030565) and human *FGF23* (NM_020638) were bought from SABiosciences. All other primers were synthesized by Integrated DNA Technologies (Table S1).

Microarray analyses were performed in the Microarray Core Facility of University of Texas Southwestern Medical Center at Dallas, using total RNA extracted from the calvaria of 3-week-old mice. GeneChip Mouse Genome 430 2.0 Array (Affymetrix) was employed in the microarray analyses, following the manufacturer's instructions. Data analyses were performed using GeneSpring software (Agilent Technologies). The microarray results were uploaded to MAGE-TAB ArrayExpress database (accession number E-MTAB-772).

### Serum biochemistry

Serum phosphorus was measured using the phosphomolybdate-ascorbic acid method, as previously described [57]. Serum calcium was measured using a colorimetric calcium kit (Stanbio Laboratory). The serum FGF23 and PTH levels were measured using a full-length FGF23 ELISA kit (Kainos Laboratories) and a mouse intact PTH ELISA kit (Immutopics). Serum 1,25(OH)_2_D_3_ was measured using a 1,25 Dihydroxy Vitamin D EIA Kit (Immunodiagnostic Systems). Blood urea nitrogen (BUN) was measured using a BUN Reagent Kit (BQ Kits).

### Generation of recombinant mouse FAM20C

The recombinant mouse FAM20C was expressed by insect cells using a Bac-to-Bac baculovirus expression system (Invitrogen). Briefly, the N-terminal of mouse FAM20C (signal peptide removed) was fused with a baculovirus signal sequence gp67 (envelope glycoprotein), 6xHis (tag), SUMOstar (Small Ubiquitin-like Modifier), and a TEV (Tobacco Etch Virus) cleavage site. The fusion gene was inserted into the pFastDual vector (Invitrogen) downstream of a polyhedrin promoter, and a GFP cDNA was inserted downstream of a P10 promoter serving as a baculovirus indicator. The construct was transformed into DH10Bac E. Coli cells (Invitrogen), in which the fusion gene was introduced into BacMid via homologous recombination. The BacMid was extracted from DH10Bac E. Coli cells and transfected into Sf21 insect cells (Invitrogen) to produce the baculovirus. The insect cells infected with the baculovirus secreted the recombinant FAM20C into the SFX-insect cell culture medium (Hyclone). After two rounds of scale-up, the cell culture medium was collected and subjected to a one-step Ni-NTA purification. Turbo-TEV (Eton Bioscience) was used to release mouse FAM20C from the fusion protein, and reverse Ni-NTA purification was performed to remove Turbo-TEV, His tag, and SUMOstar.

### Cell culture and in vitro gain- and loss-of-function analyses

Mouse MC3T3-E1 cells were grown in α-MEM medium (Gibco) supplemented with 10% fetal bovine serum (Hyclone) and antibiotics (Gibco); human Saos-2 cells (ATCC, osteoblasts from human osteosarcoma) were grown in McCoy's 5a medium (Gibco) supplemented with 15% fetal bovine serum; human mesenchymal stem cells (hMSC) (Lonza) were maintained in MSCGM BulletKit medium (Lonza). Cells were treated with recombinant FAM20C or the viruses when they reach 80% confluence. For the gain-of-function analyses, MC3T3-E1 cells were treated with mouse recombinant FAM20C at the concentrations of 200 ng/ml, 400 ng/ml and 800 ng/ml. For the loss-of-function studies, MC3T3-E1, Saos-2 and hMSC cells were infected with the mouse or human shRNA-lentiviruses (all from Santa Cruz) containing a mixture of three target-specific shRNA sequences against the mouse or human *FAM20C*. A lentivirus expressing the scrambled shRNA (with no specific target in the genome) served as the control virus (Santa Cruz). The infection rate was monitored by infecting these cells with another control virus (Santa Cruz) expressing the GFP indicator. After 1-week selection with 5–8 µg/ml concentrations of puromycin (Santa Cruz), the control virus with GFP showed a nearly 100% infection rate. To induce osteogenic differentiation for the MC3T3 cells and Saos-2 cells, the culture medium was supplemented with 100 µg/ml ascorbic acid, 10 mM β-glycerophosphate and 30 nM dexamethasone; the osteogenic differentiation of human MSC was induced using the Osteogenic BulletKit (Lonza) following the manufacturer's instruction. Total RNA was extracted from the cells at different time points. For the gain-of-function analyses, RNA was extracted after 3 weeks of induction. For the “shRNA knockdown” analyses, RNA was extracted at 3 days after the lentiviral infection and before the osteogenic medium (inducing the osteogenic differentiation) addition, and at 1, 2 and 3 weeks after the start of osteogenic induction. Real-time PCR was performed to evaluate the mRNA levels of the selected genes. The mineral deposition rate was determined by nodule formation and Alizarin red concentration in each well were measured using the Osteogenesis Quantitation kit (Millipore) for MC3T3-E1 cells treated with recombinant FAM20C (the gain-of-function experiments). MC3T3-E1 cells and hMSC cells infected with the FAM20C-shRNA viruses became unhealthy (showing lot of cell death) after 4-week culture. The Saos-2 cells could not survive in the osteogenic medium for longer than 2 weeks. The data collected from the culture of cells at the “unhealthy stages” were discarded.

### Statistics

The data are expressed as the mean ± SD of at least 6 individual determinations in all experiments unless otherwise indicated. We statistically evaluated the data employing ANOVA to test for any differences among the sample groups. When a difference was determined, 2-sample *t* tests were employed to evaluate all possible pairs of samples.

## Supporting Information

Table S1 Primers used for the real-time PCR (Q-PCR) analyses. Primers for real-time PCR analyses on critical genes related to biomineralization, osteoblast/osteocyte differentiation and phosphate homeostasis. The mRNA samples used for the real-time PCR were extracted from human and mouse osteogenic cells, mouse bone and mouse kidney.
(DOC)
