# Supplementary material for: Inactivation of a Novel FGF23 Regulator, FAM20C, Leads to Hypophosphatemic Rickets in Mice
Source: PLoS Genet. 2012 May 17;8(5):e1002708. doi: 10.1371/journal.pgen.1002708 (PMC3355082; doi:10.1371/journal.pgen.1002708)
Supplement: Table S1 — Primers used for the real-time PCR (Q-PCR) analyses. Primers for real-time PCR analyses on critical genes related to biomineralization, osteoblast/osteocyte differentiation and phosphate homeostasis. The mRNA samples used for the real-time PCR were extracted from human and mouse osteogenic cells, mouse bone and mouse kidney. (DOC) [file pgen.1002708.s001.doc]

**Table S1. Primers used for the real-time PCR (Q-PCR) analyses**

| Gene symbol | Genebank | Forward primer | Reverse primer |
| --- | --- | --- | --- |
| m-Axin2 | NM_015732 | GCTCCAGAAGATCACAAAGAGC | AGCTTTGAGCCTTCAGCATC |
| m-Bsp | NM_008318 | CAGAGGAGGCAAGCGTCACT | CTGTCTGGGTGCCAACACTG |
| m-Dmp1 | NM_016779 | CGCATCCCAATATGAAGACTG | GCTTGACTTTCTTCTGATGACTCA |
| h-DMP1 | NM_004407 | ACAGCAGCTCAGCAGAGAGT | TAATAGCCGTCTTGGCAGTC |
| m-Enpp1 | NM_008813 | GCTAATCATCAGGAGGTCAAG | CTGGTAGAATCCCGTCAATC |
| m-Fgf23 | NM_022657 | ACAAGGACACCTAAACCGAACAC | AGCTACTGACTGGTCCTATCACAGAA |
| m-Follistatin | NM_008046 | CCAGGCAGCTCCACTTGTGT | AGTCACTCCATCATTTCCACAAAG |
| m-Klotho | NM_013823 | CTGGCTAAGGTTCAAGTACGGAGACCTCCC | GGAGCTGAGCGATCACTAAGTGAATACGCA |
| m-Lef1 | NM_010703 | CCGAAGAGGAAGGCGATTTAGC | GGTCCCTTGTTGTAGAGGCC |
| m-Lgr5 | NM_010195 | CGGGACCTTGAAGATTTCCT | GATTCGGATCAGCCAGCTAC |
| m-NaPi2a | NM_011392 | GCCACTTCTTCTTCAACATC | CACACGAGGAGGTAGAGG |
| m-Osteocalcin | NM_007541 | CTTGAAGACCGCCTACAAAC | GCTGCTGTGACATCCATAC |
| m-Osterix | NM_130458 | CTACCCAGCTCCCTTCTCAA | CTTGTACCACGAGCCATAGG |
| m-Phex | NM_011077 | GCATGATTAACCAGTATAGCAA | GGTCTATAGGAATTGCACCTTAC |
| m-Sfrp1 | NM_013834 | CATCCATGGGGCTACAGTGA | TGGCATGGTGAGTTTTCAGG |
| m-Sfrp3 | NM_011356 | TGATGCTAGCGATTCCACTC | GTGTCTGCGGTAAACAATCG |
| m-1α(OH)ase | NM_010009 | CCGCGGGCTATGCTGGAAC | CTCTGGGCAAAGGCAAACATCTGA |
| m-24(OH)ase | NM_009996 | TGGGAAGATGATGGTGACCC | ACTGTTCCTTTGGGTAGCGT |

m-, mouse; h-, human; Axin2, axis inhibition protein 2; Bsp, bone sialoprotein; Dmp1/DMP1, dentin matrix protein 1; Enpp1, ectonucleotide pyrophosphatase/phosphodiesterase 1; Fgf23, fibroblast growth factor 23; Lef1, lymphoid enhancer binding factor 1; Lgr5, leucine-rich repeat-containing G protein-coupled receptor 5; NaPi2a, Na(+)-dependent phosphate cotransporter 2A; Phex, phosphate regulating gene with homologies to endopeptidases on the X chromosome; Sfrp1, secreted frizzled related protein 1; 1α(OH)ase, 25-hydroxyvitamin D(3) 1-alpha-hydroxylase; 24(OH)ase, 1,25-dihydroxyvitamin D(3) 24-hydroxylase.
